# ProNGF promotes brain metastasis through TrkA/EphA2 induced Src activation in triple negative breast cancer cells

**DOI:** 10.1186/s40164-023-00463-6

**Published:** 2023-12-10

**Authors:** Julien Cicero, Sarah Trouvilliez, Martine Palma, Gaetan Ternier, Laurine Decoster, Eloise Happernegg, Nicolas Barois, Alexandre Van Outryve, Lucie Dehouck, Roland P. Bourette, Eric Adriaenssens, Chann Lagadec, Cagatay Mehmet Tarhan, Dominique Collard, Zied Souguir, Elodie Vandenhaute, Grégory Maubon, François Sipieter, Nicolas Borghi, Fumitaka Shimizu, Takashi Kanda, Paolo Giacobini, Fabien Gosselet, Nathalie Maubon, Xuefen Le Bourhis, Isabelle Van Seuningen, Caroline Mysiorek, Robert-Alain Toillon

**Affiliations:** 1 UMR9020-U1277 - CANTHER - Cancer Heterogeneity Plasticity and Resistance to Therapies, University of Lille, CNRS, Inserm, CHU Lille, Boulevard du Professeur Jules Leclercq, 59000 Lille, France; 2 Laboratoire de La Barrière Hémato-Encéphalique (LBHE), University of Artois, UR 2465, F-62300 Lens, France; 3 GdR2082 APPICOM- « Approche Intégrative Pour Une Compréhension Multi-Échelles de La Fonction Des Protéines Membranaires », Paris, France; 4 UMR-S1172, University of Lille, Inserm, CHU Lille, Équipe Développement et Plasticité du cerveau neuroendocrine, Lille Neuroscience et Cognition, 1 Place de Verdun, 59000 Lille Cedex, France; 5 University of Lille, CNRS, CHU Lille, Institut Pasteur de Lille, US 41 – UAR 2014 – PLBS, 59000 Lille, Inserm France; 6 UMR 8520 -IEMN - Institut d’Electronique de Microélectronique et de Nanotechnologie, University of Lille, CNRS, Centrale Lille, Junia, University Polytechnique Hauts-de-France, 59000 Lille, France; 7 LIMMS/CNRS-IIS IRL2820, The University of Tokyo, Tokyo, Japan; 8 CNRS, IIS, University of Lille SMMiL-E Project, 59000 Lille, COL France; 9 HCS Pharma, 59120 Loos, France; 10 Université Paris Cité, Centre National de La Recherche Scientifique (CNRS), Institut Jacques Monod, 15 rue Hélène Brion, 75013 Paris, France; 11 Department of Neurology and Clinical Neuroscience, Yamaguchi University Graduate School of Medicine, Ube, Japan

**Keywords:** proNGF, TrkA, EphA2, Src, Brain metastasis, Breast cancer

## Abstract

**Background:**

Triple-Negative Breast Cancer is particularly aggressive, and its metastasis to the brain has a significant psychological impact on patients' quality of life, in addition to reducing survival. The development of brain metastases is particularly harmful in triple-negative breast cancer (TNBC). To date, the mechanisms that induce brain metastasis in TNBC are poorly understood.

**Methods:**

Using a human blood–brain barrier (BBB) in vitro model, an in vitro 3D organotypic extracellular matrix, an ex vivo mouse brain slices co-culture and in an in vivo xenograft experiment, key step of brain metastasis were recapitulated to study TNBC behaviors.

**Results:**

In this study, we demonstrated for the first time the involvement of the precursor of Nerve Growth Factor (proNGF) in the development of brain metastasis. More importantly, our results showed that proNGF acts through TrkA independent of its phosphorylation to induce brain metastasis in TNBC. In addition, we found that proNGF induces BBB transmigration through the TrkA/EphA2 signaling complex. More importantly, our results showed that combinatorial inhibition of TrkA and EphA2 decreased TBNC brain metastasis in a preclinical model.

**Conclusions:**

These disruptive findings provide new insights into the mechanisms underlying brain metastasis with proNGF as a driver of brain metastasis of TNBC and identify TrkA/EphA2 complex as a potential therapeutic target.

**Supplementary Information:**

The online version contains supplementary material available at 10.1186/s40164-023-00463-6.

## Background

Triple-negative breast cancer (TNBC), a breast cancer subtype characterized by lack of estrogen and progesterone receptor expression and absence of EGFR-2 (HER2/erbB2) overexpression, accounts for 15–20% of all breast cancers and most commonly affects young women under the age of 45. Unfortunately, approximately 30–40% of TNBC patients develop brain metastases, which have a particularly poor prognosis with a median survival of less than 5 months [1, 2]. These brain metastases not only lead to death, but also cause severe cognitive complications that negatively affect both the physical and psychological well-being of patients, significantly reducing their overall quality of life (loss of visual acuity, behavioral disorders, etc.) [3]. To date, no targeted therapy exists, highlighting the critical need to identify key molecular players that promote brain tropism and secondary tumor formation in the brain microenvironment. Differential transcriptomic studies of primary and/or secondary tumor samples have established predictive metastatic signatures [4–7]. The development of brain metastasis involves, among many other steps in the metastatic cascade, the interaction and transmigration of cancer cells across the blood–brain barrier (BBB) and the establishment of a supportive environment for tumor growth in the brain parenchyma [8]. These steps require dynamic interactions between cancer cells and the brain microenvironment [9].

In this context, we have shown that the tyrosine kinase receptor TrkA, was overexpressed in up to 20% [10] of TNBC cases and was involved in both tumorigenesis and the metastasis in vitro and in vivo [11–14]. TrkA is the high-affinity receptor of nerve growth factor (NGF) [15, 16] and in TNBC, both NGF and its precursor the proNGF have been found to increase invasion and migration through TrkA-mediated mechanisms [12, 17]. These two growth factors act through TrkA phosphorylation and underlying signaling pathway [14, 17]. However, for the first time, we have shown that NGF and proNGF can also activate TrkA-independent signaling through [18] the formation of TrkA/CD44v3 [11, 19] and TrkA/EphA2 [20] receptor complexes respectively. Notably, proNGF is the common form of NGF in the brain [21]. Individually, brain metastasis has been linked to TrkA, EphA2 and its downstream signaling partner Src [14, 22, 23]. The aim of this study was to investigate the involvement of proNGF and the TrkA downstream activation pathways in the development of the TNBC cell metastatic process. By mimicking the last key steps of brain metastasis using a human BBB in vitro model, in vitro organotypic matrix, ex vivo mouse organ section cultures and mouse xenograft experiments, we show that proNGF promotes brain metastasis of breast cancer cells through the formation of the TrkA/EphA2 complex.

## Methods

### Cell culture and transfections

MDA-MB-231 and BT549 breast cancer cells were obtained from the American Type Culture Collection (ATCC, Rockville, MD, USA) and were incubated in a humidified incubator (37 °C, CO_2_ 5%) in Minimum Essential Medium Eagle (MEM, Gibco™) supplemented with 10% (v/v) inactivated fetal bovine serum (FBS, Life Technologies), penicillin (40 IU/mL, Sciencell), streptomycin (40 IU/mL, Sciencell), gentamicin (50 mg/mL, Gibco™), Zell shield^®^ (1X, Minerva-Biolabs^®^), and MEM supplement nonessential amino acid (NEAA 1X, Gibco™). ProNGF (cleavage resistant) from Alomone Labs was dissolved in filtered distilled water and used at a concentration of 0.5 nM on TNBC cells.

Endothelial cells were differentiated from CD34 + hematopoietic stem cells isolated from umbilical cord blood according to the method reported by Pedroso et al. [24]. The protocol was approved by the French Ministry of Higher Education and Research (reference: CODECOH DC2011-1321) and by the local investigational review board (Béthune Maternity Hospital, Beuvry, France). Briefly, CD34 + cells were seeded at a density of 100 000 cells/cm2 in 24-well plates (Corning Inc.) and were incubated (37 °C, 5% CO2) in Endothelial Cell Medium (ECM, Sciencell) with 20% (v/v) heat inactivated FBS and Vascular Endothelial Growth Factor (VEGF, 50 ng/mL, PreproTech Inc.) for 20 days. Then, endothelial cells were cultured in gelatin-coated Petri dishes (100 mm diameter, Corning) in ECM-5 medium containing ECM medium supplemented with 5% (v/v) FBS, 1% (v/v) Endothelial Cell Growth Supplement (ECGS, Sciencell) and gentamicin (50 μg/mL, Biowest).

Human brain pericytes were provided by Professor Takashi Kanda (Department of Neurology and Clinical Neuroscience, Yamaguchi University Graduate School of Medicine, Ube, Japan). Human brain pericytes (Cosmo Bio Co., Ltd, Japan) are seeded in Petri dishes (100 mm diameter, Corning) coated with collagen I (100 μg/mL, Corning) and maintained in Dulbecco’s Modified Eagle Medium (DMEM, Life technologies) containing D-glucose (4.5 g/L) supplemented with 10% (v/v) FBS, penicillin (40 IU/mL), streptomycin (40 IU/mL) and L-glutamine (2 mM, Merck).

TrkA and TrkA KD overexpressing breast cancer cells were generating by electroporation using Amaxa Nucleofector technology (Lonza, Switzerland) according to the manufacturer’s instructions. For the establishment of stable lines, cells are maintained under selection pressure with geniticin (50 mg/mL, Gibco™).

For Src FRET biosensor experiments [25], cancer cells were seeded in 100 mm diameter dishes (24 h, 37 °C, CO_2_ 5%). The plasmid (4 µg) is diluted in a buffer solution (500 µL, JetOptimus^®^ Buffer) and a transfection agent was added (5 µL, 10 min, TA, JetOptimus^®^ reagent). The mixture was incorporated into the cell medium (6 h, 37 °C, CO_2,_ 5%). In the case of RNA interference (siRNA), a solution containing medium (MEM; 10% FBS; 500 µL), Interferin (20 µL) and siRNA (40 µM; 1 µL) was incorporated into the cell medium after incubation (20 min; RT). The cells were incubated for 36 h at 37 °C.

### Establishment of the human BBB in vitro model

According to the protocol of Vandenhaute et al. [26], after a treatment with Ethylene-diamine-tetra-acetic acid solution/trypsin (EDTA 0.025% (w/v), 1X Trypsin, Biowest), Human endothelial cells are seeded at 70 000 cells/cm2 rate on Matrigel® (Growth Factor Reduced, Corning) coated insert filters (3 μm pores, Corning) and cultivated in ECM-5 medium (37 °C, 5% CO2). Pericytes were treated with the EDTA/trypsin solution and then seeded at a 13 000 cells/cm2 rate on collagen I (100 μg/mL)-coated 1-well plates (Corning). After 6 days of culture alone, the endothelial cells were transferred on top of the pericyte culture and incubated and cultivated for an additional 6 days (37 °C, 5% CO2). After 6 days of coculture, the required time for the induction of BBB properties by pericytes, endothelial cells were then called brain-like endothelial cells (BLECs) and had the properties of brain endothelial cells. The human BBB model was then used for experiments.

### Endothelium permeability assay to Lucifer Yellow

The integrity of the BLEC monolayer was evaluated by measuring the diffusion of a BBB integrity marker that faintly crosses the BBB. To do so, the inserts containing the BLEC monolayer or only the coated insert (Matrigel^®^) were transferred into 12-well plates containing Ringer-HEPES (RH) buffer (NaCl 150 mM, KCl 5.2 mM, CaCl2 2.2 mM, MgCl2 0.2 mM, NaHCO3 60 mM, HEPES 5 mM, glucose 2.8 mM; pH 7.4). The integrity marker Lucifer yellow (LY), diluted in RH buffer (50 μM, Sigma‒Aldrich), was then added to the upper compartment. Every 20 min up to one hour, the insert filter was transferred into another well filled with RH buffer. At the end of the kinetic evaluations, the fluorescence intensity of aliquots from the initial solution, the lower compartments at each time point of the kinetics, and the upper compartment was quantified using a multiplate reader (425/538 nm, Synergy H1, BioTek Instruments). To obtain a concentration-independent parameter, the clearance principle was used. The slopes of the clearance curves for the insert with endothelial cells and the coated filters were PSt and PSf, respectively, where PS = permeability x insert surface area. The endothelial permeability (PSe) was calculated according to the formula 1/PSe = 1/PSt-1/PSf. The endothelial permeability coefficient (Pe, expressed in cm/min) was obtained by dividing the value of PSe by the insert filter surface area (1.12 cm2).

### Cancer cell trans-BBB migration experiments

The trans-BBB migration experiment is performed according the protocols of Drolez et al. and Vandenhaute et al*.* [26, 27]. Briefly, cancer cells were loaded with Green-CellTracker™ (5-chloro-methylfluorescein-diacetate (CMFDA), 2.5 μg/mL, Invitrogen) dye according to the manufacturer’s instructions. Then, the cells were rinsed with phosphate-buffered saline (PBS, NaCl 8 g/L, KCl 0.2 g/L, KH_2_PO_4_ 0.2 g/L, NaHPO_4_ 2.86 g/L, pH 7.4) and treated with EDTA solution (5 mM, 10 min, 37 °C) before being mechanically detached and resuspended in high glucose DMEM supplemented with 1% (v/v) FBS. Cancer cells were placed in contact with endothelial cells (80 000 cells/filter, 16 h, 37 °C). ProNGF (0.5 nM, Alomone-Labs) was added to the abluminal compartment. At the end of the incubation time, the filters were rinsed (DMEM high glucose) to remove nonadherent cells and fixed with paraformaldehyde (PAF 4% (w/v), 10 min, in darkness, 20 °C). The nuclei were counterstained with Hoechst 33258 (1 mM, 10 min, in darkness), and filters were separated before being mounted on slides and coverslipped with fluorescent mounting medium (Dako^®^). Images were acquired using a Plan Fluor 20x/0.45 air objective on an inverted microscope (Eclipse TiU, Nikon) with the accompanying Nikon software (NIS element AS 4.60). Transmigrating cells were counted manually on the entire filter surface (100 fields per filter).

### Brain & liver slices assay

Brains and livers from 6- to 10-week-old female C57BL/6 mice (Jackson Laboratories) were extracted in ice-cold dissection medium (MEM 75% (v/v), Hanks’ balanced salt solution (HBSS) 25% (v/v), NEAA 1X, penicillin 40 IU/mL, streptomycin 40 IU/mL). Brains were fixed and stabilized by water-resistant glue (SuperGlue, Loctite^®^) on a vibratome stage. The brain tissue was sectioned horizontally at a thickness of 300 μm by using a vibratome (Leica, VT1200S). Brain sections were transferred to filtered inserts (SARSTEDT) in 6-well plates (Greiner Bio-one) with incubation medium (MEM 50% (v/v), HBSS 25%, NEAA 1X, penicillin 40 IU/mL, streptomycin 40 IU/mL, ZellShield 1X) in the bottom well (1 mL, 37 °C, CO_2_ 5%, ovn). Cancer cells were loaded with Green-CellTracker™ (CMFDA) dye and then incubated in Matrigel^®^ (600 000 cell, 37 °C, CO_2_ 5%) and placed on a sterile plastic spacer (4 mm diameter) for 1 h. Then, the spacer was removed, and the tumor cells were incubated in contact with the organ Sect. (72 h, 37 °C, CO_2_ 5%). The interface between the tumor cells and the tissue section was observed using a Plan Fluor 10x/0.30 objective on a laser scanning confocal microscope (LSM 880, Zeiss). The number of cancer cells invading the area were analyzed manually with ImageJ software (v2.3.0/1.53f).

### 3D organotypic extracellular matrix

Breast cancer cells were seeded in brain or liver organotypic extracellular matrix (BIOMIMESYS^®^ hydroscaffold, HCS Pharma) and incubated in a humidified chamber (37 °C, CO_2_ 5%, 3 weeks). Then, all cells were fixed (PFA 4% (w/v), 10 min, 20 °C) and labeled with Hoechst 33258 (10 min) and Alexa Fluor™ 488 Phalloidin (2 h, 20 °C, ThermoFisher). The microscopy images were acquired using an automated ImageXpress^®^ Micro Confocal microscope (Molecular Devices) in confocal mode with a Plan Apo Lambda 20x/0,45 objective. The cell number and colony shape were segmented and quantified with both ImageJ and Imaris 9.8 (Bitplane) software.

### Western blotting

Cells were lysed in buffer (HEPES 40 mM, pH 7.5; EDTA 1 mM pH 8.0; NaCl 120 mM; 10 mM; NaPPi 50 mM NaF 50 mM; Na3VO4 1.5 mM; Triton-X100 1% (v/v); sodium lauryl sulfate (SDS) 0.1% (v/v); PMSF 1 mM; protease cocktail inhibitor 1% (v/v); glycerol 10% (v/v)) and then frozen (12 h, − 80 °C). The lysates were recovered by scraping and centrifuged (13 800 g; 10 min; 4 °C). Protein extracts in Laemmli buffer (Tris HCl 63 mM; glycerol 10% (v/v); SDS 2% (w/v); β-mercaptoethanol 5% (v/v); bromophenol blue 0,025% (v/v); pH 6,8) were loaded (40 µg/well) and separated on polyacrylamide gel (agarose 10%, 180 V constant, 1h15). Proteins were transferred (Tris 25 mmol/L, glycine 192 mmol/L, methanol 15% (v/v), H2O) into PVDF membrane (100 V, 1.5 h). The membranes were saturated (1 h, 20 °C, shaking) in TBS-0.1% (v/v) Tween-20 (TBST) with 5% (w/v) BSA. Then they were incubated with the primary antibodies diluted in the saturation buffer (BSA 5% (w/v), 16 h, 4 °C, shaking). The following primary antibodies were used: anti-beta-Actin (Sigma-Aldrich, A2066, rabbit), anti-pTrkA (Tyr674/675, Cell Signaling, 9141, rabbit), anti-TrkA (Cell Signaling, 2510, rabbit), anti-Src (Cell Signaling, 2123, rabbit), anti-pSrc (Tyr416, Cell Signaling, 2021, rabbit). After washing (TBST 0.1% (v/v), 5 × 5 min, 20 °C), the membranes were incubated with the secondary antibody (HRP-linked Anti-rabbit, Goat, 1/5000, 7074, Cell signaling) diluted in TBST (0.1% (v/v)). After the washing step, the chemiluminescence reaction was carried out (West Pico, ThermoScientific). Photons were detected in the darkroom using a camera (FUJIFILM LAS-4000), and the results were processed using ImageJ software.

### Immunocytochemistry

Cells were seeded on type I collagen coated (100 µg/mL) compartmentalized slides (Thermo Fisher Nunc™ Lab-Tek™). At the end of the experiment, the cells were fixed with paraformaldehyde solution (PFA 4% (w/v), pH 7.4; 10 min, 4 °C). The cells are permeabilized with a solution of Triton-X100 (0.3% (v/v), 2 × 5 min, 20 °C, sigma). Subsequently, wells were treated with saturation buffer (NDS 5% (v/v), BSA 1% (w/v), 1 h, 20 °C) before the primary antibody incubation (4 °C, ovn). The following primary antibodies were used: anti-Claudin-5 (Invitrogen, 34–1600, rabbit), anti-EphA2 (R&D systems, AF3035, goat), anti-TrkA (Alomone, ANT-018, rabbit), anti-Src (Cell signaling, 2123, rabbit), anti-pSrc (Tyr416, Cell signaling, 2021, rabbit) and anti-VE-cadherin (Invitrogen, 36–1900, rabbit). After washing step (PBS, 4 × 5 min, 20 °C), appropriated AlexaFluor conjugated-secondary antibodies were incubated (1.5 h, 20 °C). The compartmentalized slides were coverslipped with the fluorescent mounting medium (Dako^®^). Labeling was visualized using Plan-Apochromat 63x/1.4 Oil objective with laser scanning confocal microscope (LSM 880, Zeiss). For colocalization experiments, the image resolution was calculated according to the Rayleigh criterion (*r* = 0.61 (λ/NA)). Then, the images were quantified with the EzColocalization plugin [28] on ImageJ software to estimate the threshold overlap score (TOS) of the different labeling.

### Proximity ligation assay (PLA)

Cells were seeded on type I collagen coated (100 µg/mL) compartmentalized slides (Thermo Fisher Nunc™ Lab-Tek™). At the end of the experiment, the cells were fixed with paraformaldehyde solution (PFA 4% (w/v), pH 7.4; 10 min, 4 °C). The cells were treated with saturation buffer (NDS 5% (v/v), BSA 1% (v/v), 1 h, 20 °C) before the primary antibody incubation (4 °C, ovn). After washing step (PBS, 4 × 5 min, 20 °C), PLA was performed using a Duolink in Situ-Red kit rabbit/goat (Sigma-Aldrich) according to the manufacturer’s protocol. Labelling was visualized using Plan Fluo 100x/1.3 Oil objective with laser scanning confocal microscope (LSM 880, Zeiss). In-house automatic script on ImageJ was computed to estimate the total number of TrkA/EphA2 red dots per cell. First, the channels were split and an appropriate background subtraction was found for each channel to enable accurate quantification. To do this, Gaussian blur, sharpen function and threshold were optimized together to enhance the noise/signal ratio. Based on fluorescence intensity, size and circularity of the particles, the PLA signals and the nuclei were quantified.

### FRET imaging in living cells

Two hours before image acquisition, the cells expressing the Src biosensor were starved in fresh MEM without phenol red containing FBS 0.1% (v/v). During image acquisition, the cells were maintained in an incubation chamber (37 °C, CO_2_ 5%) installed on a laser scanning confocal microscope (LSM 880, ZEISS). Images were acquired using a plan-apochromat 63 × /1.4 oil objective. The lasers were tuned to emit 458 nm and 514 nm wavelengths laser lines through a 470 to 500 nm bandpass emission filter (BP470–500) for ECFP detection and a 530 nm longpass emission filter (LP530) for YPET detection. For the sensitized emission method, the emission of the ECFP, FRET and YPET channels was recorded. The fluorescence emission ratios were computed using ImageJ software to address a Src activity channel. For the acceptor photobleaching method, the images in the ECFP and YPET channels were collected prior to and following acceptor fluorescent photobleaching.

### Tumor xenograft growth in Severe combined immunodeficient mice (SCID) mice

MDA-MB-231 TrkA- or TrkA KD-overexpressing cells were subcutaneously injected (3 000 000 cells/mouse) into the flanks of six-week-old female SCID mice. Three weeks after injection, the mice were randomized into 4 groups according to the different treatments. Five treatments of entrectinib (per os, 30 mg/mouse) and/or siEphA2 (subcutaneous, 7.5 µg/mouse) were given to mice in a 2- to 3-day interval. Tumor volume was quantified throughout the experiment by measuring the length (l) and width (w) and was calculated as π/6 × l × w × (1 + w)/2. The mice were sacrificed under isoflurane anaesthesia when the tumors reached a critical size. (around 4 cm^3^), and the organs were immediately removed and stored in liquid nitrogen or fixed by immersion (PFA 4% (v/v), 4 °C, ovn) and then stored for tissue clearing (PBS, 4 °C).

### Whole-mount immunostaining and tissue clearing

Perfusion-fixed whole mouse brains were processed using an adapted version of the iDISCO + protocol described previously [29, 30]. The samples were first dehydrated in a series of methanol gradients then bleached overnight with 5% H_2_O_2_ at 4 °C, and delipidated overnight in a solution of 66% dichloromethane/33% methanol at 4 °C. Two rinses in 100% methanol were performed after the bleaching and delipidation steps. The samples were then gradually rehydrated from methanol to PBS, and permeabilized and blocked-in incubation solution (PBS, 0.2% gelatin, 1% Triton X100, 0.05% sodium azide) for 4 days. The brains were subsequently incubated with primary antibodies (anti-hHLA, Goat, 1/1000, Sigma) in incubation solution for 10 days, rinsed several times in incubation solution, and further incubated with secondary antibodies (Alexa Fluor 647 anti-Goat, Donkey, 1/1000, Sigma) for 5 days. Unbound antibodies were washed out with several rinses in PBS + 1% Triton X-100. After immunostaining, the brains were dehydrated in a series of methanol gradients and incubated overnight in 66% dichloromethane/33% methanol. On the next day, the methanol was washed out by a final 1-h incubation in 100% dichloromethane. Finally, the samples were cleared by immersion in dibenzyl ether for at least 2 h in rotation. When transparency was achieved, a fresh solution of dibenzyl ether was used for storage, and the samples were kept protected from light at room temperature until imaging.

### Lightsheet imaging and analysis

Imaging of cleared tissues was performed in dibenzyl ether on an Ultramicroscope instrument (LaVision BioTec, BioImaging Center of Lille) and using either a 1.1x/0.1NA MI PLAN objective or a 4x/0.3NA LVMI-Fluor objective (LaVision Biotec). The following parameters were used: z-step was set to 2–4 µm, laser width and numerical aperture were kept at maximum, and for mosaic acquisitions a 10% overlap between tiles was configured. Acquisitions were saved as tiff sequences and converted to the Imaris file format using Imaris Converter (Bitplane).

### Scanning electron microscopy

Three-dimensional extracellular matrix samples were fixed with 1% glutaraldehyde in 0.1 M sodium cacodylate buffer overnight. After washing, the samples were treated with 1% osmium tetroxide in water in the dark for 1 h. The samples were dehydrated with a series of increasing ethanol concentration. After two pure ethanol incubations, the sample was washed with hexamethyldisilazane and then dried (20 °C, overnight). Finally, extracellular matrix was mounted on stubs and observed with a secondary electron detector in a Zeiss SEM Merlin Compact VP operating at 1 kV.

### RNA extraction from tissues

Tissues were collected in RNAse-free Precellys tubes (Ceramic beads CK14) with QIAzol lysis reagent (QIAGEN). RNA extractions were performed according to manufacturer’s instructions using the RNeasy Mini Kit (QIAGEN).

### Reverse transcription PCR (RT-PCR)

RNA was reverse transcribed using SensiFAST cDNA Kit (Ozyme) according to the manufacturer’s recommendations. PCR was performed with reversely transcribed RNA using ONE Green FAST qPCR Premix (Ozyme). The primer sequences used in this study were for: human beta-2-microglobulin (Forward 5′- CCAGCAGAGAATGGAAAGTC -3′; Reverse 5′- GATGCTGCTTACATGTCTCG -3′) and mouse beta-2-microglobulin (Forward 5′- CTGCTACGTAACACAGTTCCACCC -3′; Reverse 5′-CATGATGCTTGATCACATGTCTCG -3′). The PCR products (for human beta-2-microglobulin) were analyzed by agarose gel electrophoresis (1%) followed by ethidium bromide staining.

## Results

### ProNGF induces TrkA/EphA2 complex formation regardless TrkA phosphorylation

**Fig. 1:**
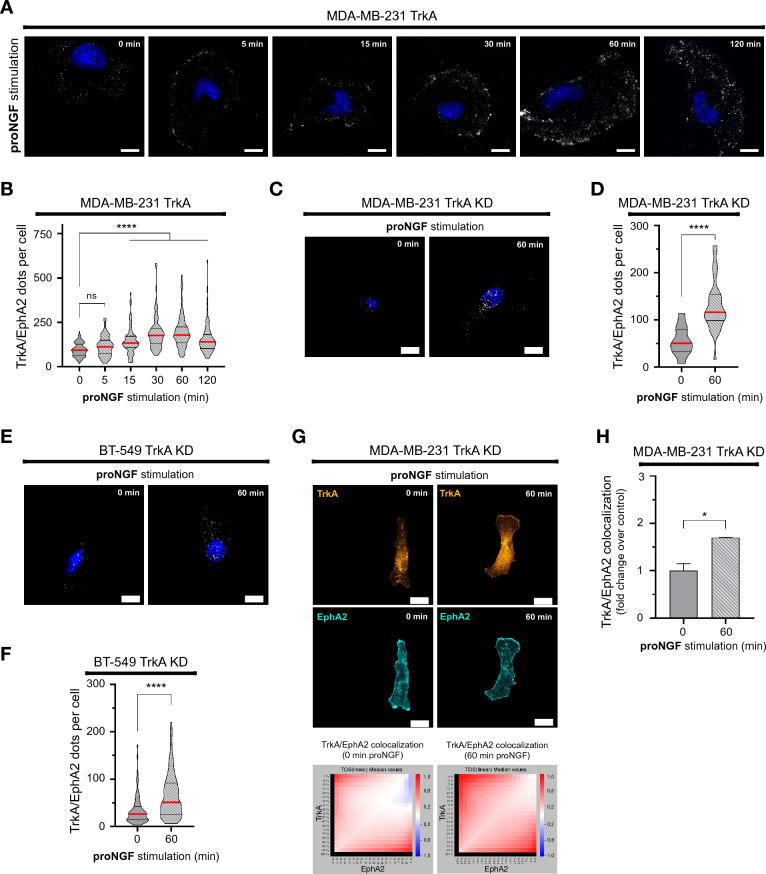
proNGF increases TrkA/EphA2 complexes in TNBC. **A** and** B** Representative overlay (**A**) and quantification (**B**) of TrkA/EphA2 PLA signal following proNGF stimulation (5, 15, 30, 60 and 120 min) in MDA-MB-231-TrkA.** C**–**F** Representative pictures of TrkA/EphA2 PLA signal following proNGF stimulation (60 min) in MDA-MB-231-TrkA-KD and BT-549 HA-TrkA KD (**C** & **E**) with respectively the associated quantification (**D** & **F**). **G** & **H** Representative confocal images of TrkA and EphA2 signal in MDA-MB-231-TrkA and TrkA/EphA2 colocalization matrix (**G**) with its quantification (**H**). Excluding experiment **G** and **H** which are performed in duplicate, data in** A**-**F** are representative of 3 independents experiments performed on 30 cells/condition each. Data in **B**, **D** and **F** are presented with violin plots demonstrating the median (red bold line), quartiles (dots lines), variability and density. Data in **H** are represented by column bar graph with SD. One-way ANOVA followed by Tukey’s test for **B**. Unpaired 2-tailed t test for **D**, **F** and **H**. *P ≤ 0.05, ****P ≤ 0.0001, ns (non-significant). For **A**, **C** and **E**, scale bar = 10 µm, and for **G**, scale bar = 15 µm

We previously showed that proNGF could induced TrkA-dependent signaling [19, 20]. Therefore, the specificity of the proNGF effect on TrkA/EphA2 complex formation was therefore quantified by proximity ligation assays. Using MDA-MB-231 cells overexpressing a TrkA protein, the effect of proNGF on TrkA/EphA2 complex formation was observed and quantified by microscopy (Fig. 1A, B). It was found that proNGF induced an increase in the TrkA/EphA2 complex at the cell membrane. This increase in TrkA/EphA2 complex formation was measured from 15 to 120 min of proNGF treatment, with a twofold increase at 60 min relative to the basal level (0 min). In addition, since proNGF is known to act through p75NTR [31], the involvement of p75NTR on proNGF-induced TrkA/EphA2 complexes was therefore assessed using siRNA (Additional file 1: Fig. S1A, B). However, invalidation of p75NTR showed no effect on proNGF-induced TrkA/EphA2 complex formation indicating that p75NTR is not involved in this mechanism. Overexpression of a TrkA kinase dead (KD) protein (triple mutant at residues Y670F-Y674F-Y675F) also confirmed that TrkA/EphA2 complex formation was independent of TrkA phosphorylation, as proNGF was still able to induce TrkA/EphA2 complex formation (Fig. 1C, D). To rule out the possibility that this phenomenon is restricted only to MDA-MB-231 cells, a second triple negative cell line (BT-549) was used (Fig. 1E, F) and transfected here with the TrkA KD expression vector. Proximity ligation assays showed that TrkA/EphA2 complex formation was also induced by proNGF treatment in these cells. The colocalization of TrkA and EphA2 was also verified by immunofluorescence and confocal laser scanning microscopy (Fig. 1G, H) using MDA-MB-231 TrkA KD cells. The images show that under the effect of proNGF treatment, the labeling of TrkA and EphA2 overlapped and appeared to be localized at the plasma membrane (cell periphery). This overlap was quantified by using a threshold overlap score (TOS) matrix (Fig. 1H) and showed a 1.7-fold increase compared to that of unstimulated cells.

Overall, these results indicated that the formation of the TrkA/EphA2 complex is dependent on proNGF. Moreover, the proNGF-induced TrkA/EphA2 complex is also independent of p75NTR and TrkA phosphorylation.

### TrkA/EphA2 complex formation induces Src activation in TNBC cells

**Fig. 2:**
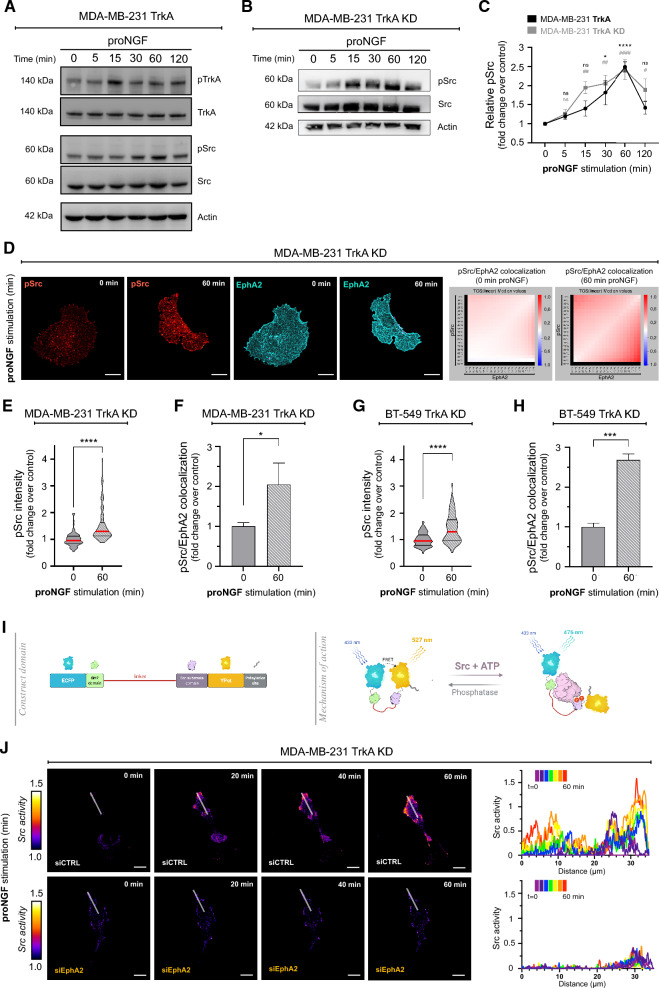
proNGF induces Src activation independently of TrkA phosphorylation in TNBC. **A**–**C** Immunoblotting from MDA-MB-231-TrkA (**A**) or MDA-MB-231-TrkA-KD (**B**) following proNGF treatment (5, 15, 30,60 and 120 min) for TrkA, Src (and their phosphorylation form) and actin with associated relative quantification of Src phosphorylated regarding to its unphosphorylated form (**C**). **D**-**H** Representative confocal images from proNGF simulated (0 or 60 min) MDA-MB-231-TrkA-KD (**D**) of Src active form (Red) and EphA2 (Blue) staining with pSrc/EphA2 colocalization matrix and respective Src activity (**E** &** F**) and EphA2/pSrc colocalization quantification (**G** & **H)** for MDA-MB-231-TrkA-KD and BT-549 KD. **I** Scheme of FRET Src reporter from Ouyang et al*.* that is characterized by an ECFP/YPET pair, an SH2 domain, a Src substrate domain and a prenylation site. (J) Emission ratio images and profiles along the indicated white lines of the ECFP/YPet-based Src biosensor in response to proNGF stimulation with or without prior transfection of siEphA2 on MDA-MB-231-TrkA-KD. Excluding experiment **B** which is performed in duplicate, data in **A** to **J** are representative of 3 independents experiments. For **D** to **H**, they are performed on 30 cells/condition each, data in **J** are realized on approximately 7 cells/condition. Data in **C** are represented by connecting line graph with mean ± SEM. Data in **E** and **G** are presented with violin plots demonstrating the median (red bold line), quartiles (thin black line), variability and density. Data in **F** and **H** are represented by column bar graph with SD. Two-way ANOVA followed by Sidak’s test for **C** with the set of conditions was compared to the corresponding 0 min time. Unpaired 2-tailed t test for **E** to **H**. * and #P ≤ 0.05, ** and ##P ≤ 0.01, *** and ###P ≤ 0.001, **** and ####P ≤ 0.0001. For D and E, scale bare = 15 µm

Since Src is over-activated in TNBC cells that metastasize to the brain and is involved in the underlying signaling pathways of TrkA, we investigated whether Src activation was due to proNGF stimulation. Src phosphorylation was first measured by western blotting in MDA-MB-231 TrkA cells treated with proNGF from 0 to 120 min (Fig. 2A). The results showed that Src was phosphorylated under the effect of proNGF, with Src activation persisting until 60 min, reaching 2.5 times the control level and then returning to an activation basal level at 120 min. Since the TrkA/EphA2 complex is formed independently of TrkA phosphorylation, we evaluated whether Src activation is also independent of TrkA phosphorylation. First, Src activation was measured by western blotting in MDA-MB-231 TrkA KD cells treated with proNGF (Fig. 2B). Thus, in MDA-MB-231 TrkA KD cells, Src phosphorylation was still induced under the effect of proNGF with activation kinetics similar to those found in cells overexpressing nonmutated TrkA (Fig. 2C). Using entrectinib, a multikinase inhibitor, it was showed that Src phosphorylation induced by proNGF was slightly decreased in MDA-MB-231 TrkA cells (Additional file 1: Fig. S2A, 2B). In addition, Src phosphorylation was measured by immunofluorescence in MDA-MB-231 TrkA KD and BT-549 TrkA KD cells. In MDA-MB-231 TrkA KD and BT-549 TrkA KD cells, the fluorescence intensity of an anti-pSrc antibody was significantly higher in proNGF-treated cells after 60 min of treatment than in the control cells without proNGF stimulation (Fig. 2D, E, G). Quantification of fluorescence intensity showed an approximately 1.5-fold increase in fluorescence for pSrc in both cell types after proNGF treatment (Fig. 2E, ). In addition, a colocalization study of pSrc and EphA2 was performed by TOS arrays and quantification was performed (Fig. 2F, H). It was observed that proNGF treatment increased colocalization of EphA2 and pSrc in both cell types. The colocalization in the proNGF-treated group was approximately twofold higher than that in the control group in MDA-MB-231 TrkA KD cells and the colocalization in the proNGF-treated group was 2.5-fold higher than that in the control group in BT-549 TrkA KD cells. Although proNGF is known to be able to activate TrkA by p75^NTR^, we demonstrated that proNGF is able to activate Src by TrkA/EphA2 independent of p75^NTR^ (Additional file 1: Fig. S3A-F). Overall, these results showed that proNGF induces Src phosphorylation in TNBCs independent of TrkA phosphorylation.

### ProNGF-induced Src is partly dependent on EphA2

To evaluate whether Src activation under proNGF stimulation is EphA2 dependent, a FRET reporter was used (22). This biosensor, whose structure is schematized in Fig. 2I, allows the ability to quantify Src activity in real time as well as visualize its localization. As shown in Fig. 2J, Src activity increased in a time-dependent manner in the presence of proNGF in MDA-MB-231 TrkA KD cells under control conditions (Fig. 2J, Additional file 2: video S1 and Additional file 3: video S2). These activation kinetics correlated with the increase in Src phosphorylation. This activation was found at the level of the plasma membrane, and more particularly, at the level of the membrane protrusion. Moreover, the inhibition of EphA2 (by targeting siRNA, Additional file 1: Fig. S2C) showed that the activation of Src by proNGF was completely suppressed. Src activation induced by proNGF was also quantified by acceptor photobleaching (AB-FRET) in MDA-MB-231 TrkA KD cells and MDA-MB-231 TrkA cells after entrectinib treatment and also with and without EphA2 expression inhibition by siRNA. The AB-FRET results showed that the proNGF-treated group exhibited a decreased donor intensity level compared to that of the control group in MDA-MB-231 TrkA KD cells, indicating Src activation. Inhibition of EphA2 restored the donor fluorescence intensity level, indicating that Src activation by proNGF was dependent on EphA2 expression (Additional file 1: Fig. S4A, B, C). In MDA-MB-231 TrkA cells, Src activation was also induced by proNGF treatment, as indicated by the decreased donor intensity level. Entrectinib, which inhibits TrkA phosphorylation, did not affect proNGF-induced Src activation. In contrast, EphA2 inhibition suppressed Src activation, and this inhibition was stronger in the presence of entrectinib (Additional file 1: Fig. S4D, E). Overall, the results of this FRET study showed that proNGF induces Src activation, which is independent of TrkA phosphorylation but dependent on EphA2.

### The TrkA/EphA2 complex is responsible for proNGF-induced increased TNBC cells transmigration through the BBB

**Fig. 3:**
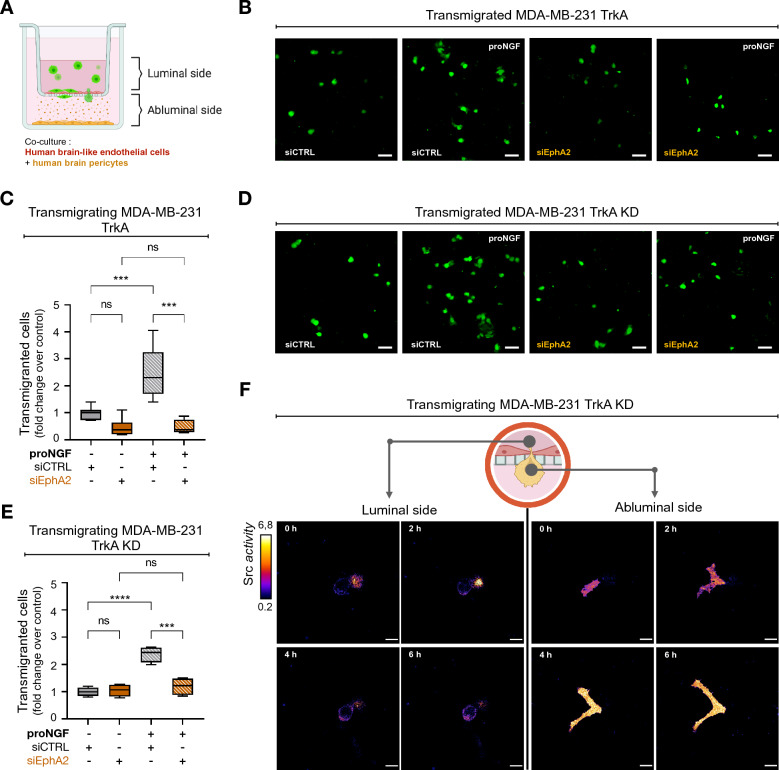
proNGF enhances trans-BBB migration through Src activation independently of TrkA phosphorylation in TNBC. **A** Schematic representation of human BBB in vitro model, this one is characterized by a luminal compartment that allow culture of human BLEC on insert filtered (3 µm) and an abluminal compartment with plated human brain pericyte. Cancer cells are load with CellTracker™ before being incubated in the upper side. **B-E** Representative overlay (**B & D**) and associated quantification (**C & E**) of CellTracker™ stained MDA-MB-231-TrkA or CellTracker™ stained MDA-MB-231-TrkA-KD (green) on the lower compartment after a 16 h trans-BBB migration. (**F**) Emission ratio images of the ECFP/YPet-based Src biosensor during MDA-MB-231-TrkA-KD trans-BBB migration under abluminal proNGF stimulation. Data in**B**-**E** are representative of 3 independents experiments with all condition realized in duplicate. Data in **C** and **E** are represented by min to max interleaved box graph with median (black line). Two-way ANOVA followed by Tukey’s test for **D** and **F**. ***P ≤ 0.001, ****P ≤ 0.0001, ns (non-significant). For B and D scale bar = 100 µm, for D and F scale bare = 15 µm

After characterizing the formation of the TrkA/EphA2 complex and the underlying activation of Src stimulated by proNGF treatment, the effects of proNGF on the underlying mechanisms of brain metastasis were investigated. First, the effects of proNGF on the transmigration of MDA-MB-231 cells through the BBB were studied using a human BBB in vitro model [32, 33]. In this model, the BBB was mimicked by establishing a coculture between brain-like endothelial cells seeded on an insert filter and human brain pericytes seeded at the bottom of the well (Fig. 3A). First, the BBB integrity was evaluated by measuring the endothelial permeability coefficient of lucifer yellow (LY) (Additional file 1: Fig. S5A), a BBB integrity marker, and visualized by immunofluorescence labeling of the adherent and tight junction proteins VE-cadherin and Claudin-5, respectively. The permeability coefficient of LY revealed that the physical integrity of the BBB was not compromised by proNGF treatments, which were added on the abluminal side to mimic brain secretion and was measured after the transmigration of MDA-MB-231 TrkA cells. The low endothelial permeability coefficient to LY was correlated with the high expression of tight junction and adherent junction proteins such as Claudin-5 or VE-cadherin continuously localized at the endothelial cell border, revealing the absence of physical BBB alteration in all experimental conditions tested (Additional file 1: Fig. S5A-C). Next, to evaluate whether proNGF stimulation modulates the capacity of cancer cells to interact with the BBB, the transmigration capacities of MDA-MB-231 TrkA and TrkA KD cells through the BBB were quantified along with the impact of EphA2 inhibition on this phenomenon. In MDA-MB-231 TrkA cells, proNGF treatment induced a 2.5-fold increase in cell transmigration across the BBB. In these cells, EphA2 inhibition did not have an effect on the transmigration capacities in the absence of proNGF stimulation; however, it reversed the increased capacity of transmigration induced by proNGF (Fig. 3B, C). Moreover, transmigration through the BBB was also measured in TrkA KD cells. Similar to what had been observed in MDA MB 231 TrkA cells, proNGF stimulation increased the cell transmigration capacity of TrkA KD cells, which was reverted following EphA2 inhibition by siRNA (Fig. 3D, E). These results suggested that TNBC cell transmigration through the BBB under proNGF stimulation depends on EphA2 but not on the TrkA phosphorylation signaling pathway.

After evaluating the effect of proNGF on transmigration, the activation of Src during transmigration was investigated. The FRET radiometric images obtained from the Src biosensor revealed that during transmigration through the BBB endothelial cells (ECs), Src was substantially activated in the proNGF-stimulated cells during passage through the endothelium (Fig. 3F, Additional file 4: video S3 and Additional file 5: video S4).

### The TrkA/EphA2 complex is involved in parenchyma invasion by TNBC cells

**Fig. 4:**
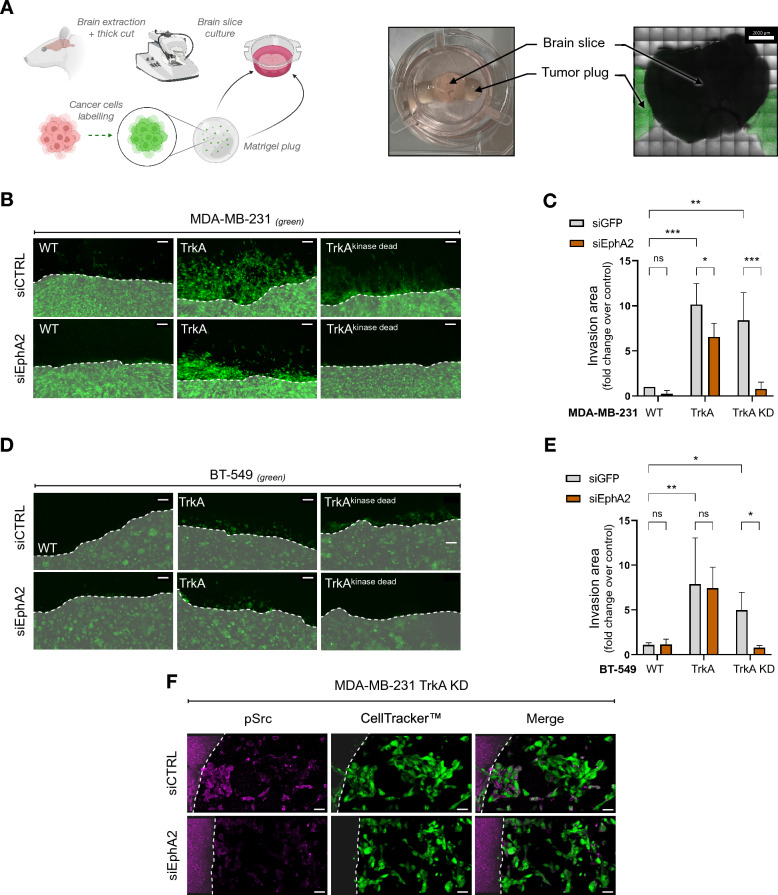
TrkA/EphA2 increases brain parenchyma invasion of TNBC. **A** Experimental approaches of ex vivo brain slices culture with TNBC. After dissection, the mouse brain is thickly sliced and cultured with the TNBC on an air/liquid interface over 72 h. **B**-**E** Representative pictures (**B** – **D**) and associated quantification **C** & **E** from the monitoring of CellTracker™ stained MDA-MB-231 WT/TrkA/TrkA KD or CellTracker™ stained BT-549 WT/TrkA/TrkA KD (green) during mouse brain slices invasion. **F** Overlay about Src activation (pink) in CellTracker™ stained MDA-MB-231-TrkA-KD (green) with or without prior siEphA2 transfection. Data in **B**-**E** are representative of 3 independents experiments with all condition realized in duplicate. Data in **F** were performed in duplicate for each condition. Data in **C** and **E** are presented as a column bar graph with SD. Two-way ANOVA followed by Tukey’s test for **C** and **E**. *P ≤ 0.05, **P ≤ 0.01, ns (non-significant). For **B**, **D** and **F**, the Matrigel® plug is highlighted by a whitish area delimited with dotted lines. For **B** and **D** scale bar = 100 µm, and **F**, scale bar = 30 µm

After transmigration through the BBB ECs, TNBC cells interact and invade the brain parenchyma. To investigate this phenomenon, the parenchyma invasion capability of cancer cells was measured using a brain section culture system (Fig. 4A). In this system, TNBC cells seeded in Matrigel® plugs were attached to mouse brain sections and cocultured for up to 3 days (the dotted line indicates the limit of the Matrigel^®^ plug). The invasion capacity of the brain parenchyma by TNBC MDA-MB-231 cells overexpressing TrkA or TrkA KD was quantified by measuring the area infiltrated by the cancer cells in the brain tissue. MDA-MB-231 TrkA cells showed a tenfold increase in brain parenchyma invasion capacity compared to that of MDA-MB-231 wild-type (WT) cells. Interestingly, TrkA KD cells also invaded the brain parenchyma. However, when EphA2 expression was inhibited, the ability to invade the brain parenchyma was significantly reduced in TrkA cells and completely abolished in TrkA KD cells (Fig. 4B, C). The results of the second TNBC cell line (BT-549) confirmed the results obtained with the MDA-MB-231 cell line (Fig. 4D, E), although the invasive capacity of these cells was lower. Taken together, these results show that TrkA phosphorylation-dependent and -independent pathways play an essential role in tumor cell invasion into brain tissue, and can mutually compensate when either is impaired. Interestingly, it was also observed that Src phosphorylation, which was induced in breast cancer cells that were in contact with the brain parenchyma, was suppressed when EphA2 expression was inhibited by siRNA in MDA-MB-231 cells (Fig. 4F). In addition, in our previous study, it was observed that MDA-MB-231 TrkA cells exhibited liver tropism, and that EphA2 inhibition did not block liver metastasis [20]. To further understand cancer cell tropisms, liver sections were used to evaluate the invasion capacities of MDA-MB-231 TrkA KD cells (Additional file 1: Fig. S6A, B). In contrast to brain invasion, liver invasion by MDA-MB-231 TrkA KD cancer cells was not impaired following EphA2 inhibition.

### The TrkA/EphA2 complex increases the clonogenic growth of TNBC cells in a 3D culture system with a mimetic brain matrix

**Fig. 5:**
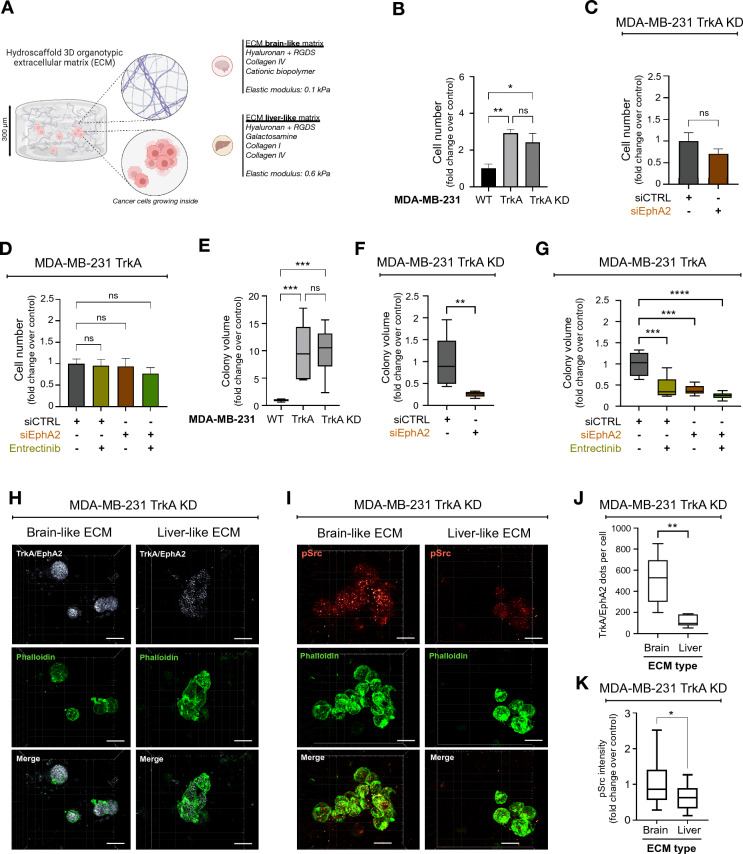
TrkA/EphA2 increases TNBC persistence in brain-like ECM. **A** Schematic representation of in vitro brain-like and liver-like 3D ECM (from BIOMIMESYS^®^) where MDA-MB-231 cells are maintained. **B**-**G** According to the TrkA overexpression status in MDA-MB-231, cell number (**B** & **C**) and colonies volumes (**E**–**G**) are quantified after entrectinib and/or EphA2 targeting siRNA of 21 days culture in 3D organotypic ECM. (**I** and **K**) Representative pictures (**H**) and associated quantification (**J**) of TrkA/EphA2 PLA signal inside MDA-MB-231-TrkA-KD incubated in brain-like or liver-like ECM. **I** and **K** Representative confocal images (**J**) and associated quantification (**L**) of phosphorylated Src (active form) signal in MDA-MB-231-TrkA-KD incubated in brain-like or liver-like ECM. Data in **B**-**G** are representative of 2 independents experiments performed in triplicate. These data H to K are from one replicate experiment performed on approximately 30 cells for each condition **C**. Unpaired 2-tailed t test for **B, C**, **E**, **F**, **J** and **K**. Two-way ANOVA followed by Tukey’s test for **D** and **G**. *P ≤ 0.05, **P ≤ 0.01, ***P ≤ 0.001, ****P ≤ 0.0001, ns (non-significant). For **H** and **J** scale bar = 20 µm

**Fig. 6:**
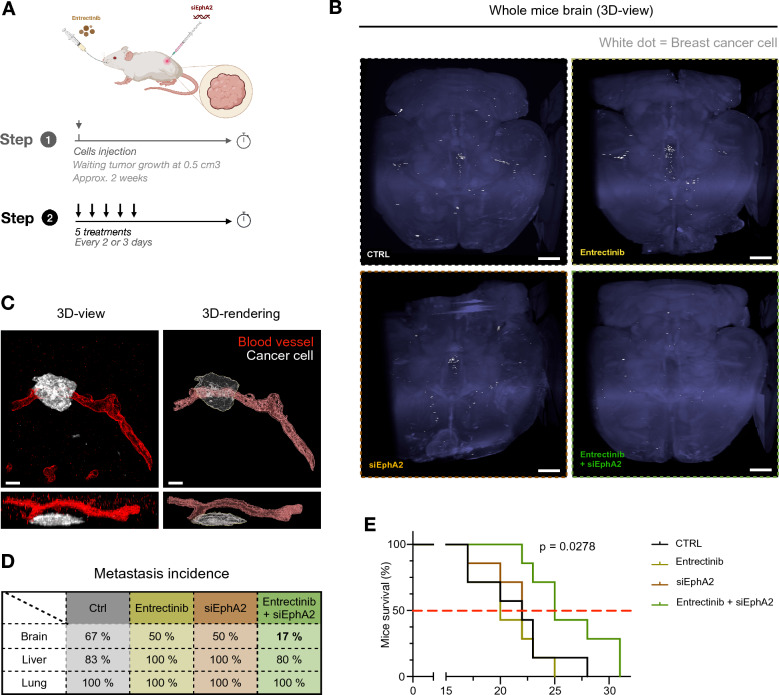
Combinatorial treatment with TrkA- and EphA2- targeting inhibitor delays tumor growth and survival and block the brain metastasis. **A** After tumor relapse from MDA-MB-231 HA-TrkA were xenografted in SCID mice treated with Entrectinib and/or EphA2-targeting siRNA 5 times every 2–3 days. **B** Cleared brain from control xenografted mice was sliced and labeled for laminin (blood vessel, red) and human HLA (MDA-MB-231-TrkA, white). **C** 3D projection of solvent-cleared brain (blue, autofluorescence) dissected from xenografted SCID mice with MDA-MB-231-TrkA revealed by human HLA immunostaining (white). **D** Detection of metastatic MDA-MB-231-TrkA (by RT-PCR for the human microglobulin expression) in brain, liver and lung from xenografted SCID mice treated with Entrectinib and/or siEphA2. **E** Survival of mice xenografted with survival median (red dotted line). All data are derived from the same experimental approach detailed in **A**. For **E**, the experiment was performed with 7 mice/group. At the end of this experiment, 1 mouse/group was used for **B** and **C**, and 6 mice/group were used for D. Data in E are represented by a staircase graph with median (red dotted line). For **E**, Mantel-Cox Log-rank analysis was realized, P-value shows the comparison between CTRL and combinatorial treatment. For **B**, scale bar = 1 mm, and scale bar = 30 µm for **C**

Cancer cells had the ability to invade the mouse brain parenchyma, as we observed in Additional file 6: video 5. Nevertheless, the duration of the brain section culture system experiments was too short to evaluate whether the cells could survive and develop colonies (micrometastases) in the brain parenchyma. Thus, clonogenic growth was measured in a 3D culture system using a matrix that mimics that human brain parenchyma in composition and stiffness (3D BIOMIMESYS® Brain) (Fig. 5A, Additional file 1: Fig. S7A). To assess whether the observed biological effects are specific to brain tropism, we also used human liver matrices that mimic the liver (3D BIOMIMESYS® Liver), another preferential metastatic site of TNBC cells. In these 3D systems, cells were double labeled using Hoechst 33,258 and phalloidin to allow the visualization of cell shape and colonies (Additional file 1: Fig. S7B). In a brain-like matrix, the number of MDA-MB-231 TrkA cells was 3 times higher than that of wild-type MDA-MB-231 cells after 21 days of culture (Fig. 5B), which was not the case in the liver-like matrix (Additional file 1: Fig. S7C). Next, the role of EphA2 and/or TrkA phosphorylation was investigated. Using MDA-MB-231 TrkA KD cells, we observed that the number of cells in the brain-like or liver-like matrix was not significantly different when EphA2 expression was inhibited by siRNA (Fig. 5C, Additional file 1: Fig. S7D). Moreover, in MDA-MB-231 TrkA cells, neither the inhibition of TrkA kinase activity by entrectinib, the inhibition of EphA2 or the combination of these two treatments had a significant effect on the number of cells in the brain and liver matrices (Fig. 5D, Additional file 1: Fig. S7E). Next, the clonogenic growth was evaluated by quantifying the number of colonies in the two matrices. Indeed, in both matrices, the number of colonies was up to tenfold higher in MDA-MB-231 TrkA cells than MDA-MB-231 wild-type cells in brain-like and liver-like matrices (Fig. 5E, Additional file 1: Fig. S7F). In addition, EphA2 inhibition in MDA-MB-231 TrkA KD cells only significantly decreased the number of colonies in the brain-like but not in the liver-like matrix (Fig. 5F, Additional file 1: Fig. S7G). Interestingly, in the brain-like matrix, the number of the MDA-MB-231 TrkA colony was decreased not only following EphA2 inhibition or entrectinib treatment but also following the combined treatment (Fig. 5G). These results were not observed in the liver-like matrix (Additional file 1: Fig. S7H). To further elucidate the molecular mechanisms, the presence of TrkA/EphA2 complexes and Src activity (measured by its phosphorylation) were also assessed in brain-like and liver-like matrices using TrkA kinase dead cells. According to the results of a proximity ligation assay (PLA), the number of TrkA/EphA2 complexes in MDA-MB-231 TrkA KD was 4 times higher in the brain-like matrix than in the liver-like matrix (Fig. 5H, J). In these TrkA KD cells, compared to the liver-like matrix, the brain-like matrix showed an increase in Src phosphorylation, which was positively correlated with the number of TrkA/EphA2 complexes (Fig. 6I, K).

The results obtained using organotypic 3D extracellular matrices confirmed that MDA-MB-231 TrkA cells had an increased ability to growth in brain-like matrices compared to that in liver-like matrices. In addition, the colony size was larger in TrkA-overexpressing cells. This increase in colony formation was dependent on the phosphorylation of TrkA and EphA2 in the brain-like matrix but not in the liver-like matrix.

### The TrkA/EphA2 complex decreases survival and increases brain metastasis in an in vivo TNBC cell xenograft model

After showing that proNGF increased transmigration of MDA-MB-231 TrkA cells across the BBB and the invasion and development of cells in the brain parenchyma via TrkA/EphA2, we investigated the ability of proNGF to induce brain metastasis using an in vivo preclinical model. For this purpose, MDA-MB-231 TrkA cells were xenografted into the flanks of severe combined immunodeficient (SCID) mice. Tumor growth was measured as well as the presence of brain metastasis (Fig. 6A, Additional file 1: Fig. S8A). First, tumor growth was assessed after different treatments that suppressed TrkA kinase activity (entrectinib), EphA2 expression (by siRNA) or both (Additional file 1: Fig. S8A). where combined treatment can delay tumor growth, treatments alone have no effect on this. Once the tumor has reached its critical size, the mouse is sacrificed to study the metastatic spread of tumor cells. Using iDISCO + , a method for immunolabeling-enabled three-dimensional imaging of solvent-cleared organs, brain micrometastases were visualized (Fig. 6B). In mice treated with the combination treatment (entrectinib and siEphA2), we observed almost no brain metastases. After sectioning the brains of untreated mice, we observed that metastatic cells were surrounding the blood vessels that led into the brain parenchyma (Fig. 6C). Confirmed by RT‒PCR quantification, we observed that brain metastasis occurred in 67% of the mice in the control group, while 50% of the mice in the entrectinib or siEphA2 groups developed brain metastasis. The combination of TrkA and EphA2 inhibition reduced the occurrence of metastasis to 17% (Fig. 6D). Moreover, this combination treatment increased the median survival of xenografted mice from 19 to 25 days (Fig. 6E). Thus, the in vivo data corroborate those obtained in vitro (BBB model, BIOMIMESYS® organotypic matrix) and ex vivo (cell culture in the presence of murine brain and liver sections).

## Discussion

In this study, we confirmed that proNGF induces the formation of the TrkA/EphA2 complex in TNBCs independently of TrkA phosphorylation. Furthermore, we showed that proNGF-induced Src phosphorylation is dependent on the TrkA/EphA2 complex. From a mechanistic point of view, we showed that proNGF is involved in brain metastasis through this complex. In addition, proNGF promotes transmigration across the BBB, invasion into the brain parenchyma and growth of TNBCs cells in extracellular matrices that mimic the brain. These findings correlate with Src activation, and brain tropism in vivo can be blocked by co-inhibition of TrkA and EphA2. For the past few decades, the incidence and death of breast cancer kept increasing worldwide and the development of metastasis is particularly harmful in breast cancer, even more so, in TNBC [34, 35]. The level of TrkA found in these cancer types is comparable to levels of other receptor tyrosine kinases, in particular Met, which is known to induce metastasis. Met amplification is 1–3% in lung adenocarcinoma [36] and reaches 20% in the case of concomitant EGFR mutation [37]. Interestingly, the oncogenic activity of growth factor receptors is related to their kinase activity [10, 38]. Thus, many therapies for brain metastasis are kinase inhibitors that target receptor tyrosine kinases (RTKs) or downstream signaling molecules [39]. In the case of TrkA, lestaurtinib, larotrectinib and entrectinib have been developed. However, the efficiencies of all three inhibitors are moderate, and only a limited benefit is shown in the context of TrkA oncogenic fusions, which reflects the extent of oncogene dependence on kinase activity. In breast cancer, these oncogenic fusions represent less than 1% of cases [40]. In our study, TrkA overexpression likely acted through its kinase activity to a certain extent. TrkA kinase activity is known to enhance the aggressiveness and metastasis of TNBC [38] and other breast cancer subtypes, such as HER2-positive breast cancer [41]. Interestingly, our results demonstrated that TrkA tyrosine kinase-independent pathways are also involved. Indeed, we demonstrated the recruitment of the EphA2 receptor under the effect of proNGF, as previously shown [20]. Interestingly, EphA2 is also overexpressed and associated with a decrease in disease-free survival and overall survival in TNBC patients [42]. In cancer, EphA2 undergoes an oncogenic switch and a loss of dependence on its ligand Ephrin A1. Moreover, the expression levels of EphA2 and Ephrin A1 are inversely correlated. EphA2 is thus, by its transactivation, involved in many oncogenic processes, such as tumor cell proliferation, survival and metastasis [43–45], and is associated with a poor prognosis in TNBC patients [42]. The present work showed that Src activation through the TrkA/EphA2 axis is critical for metastasis of TNBC cells to the brain, hence the relevance of conducting studies in this field to effectively block this pathway in TNBC cells and reduce the incidence of brain metastases. Src signaling pathways are mediated by membrane proteins, including integrins, growth factor receptors, and EPH family receptors. With these multiple key players as targets, Src is known to not only promote the proliferation but also the migratory and invasive capacities of cancer cells, which leads to increased metastatic spread [46]. Our results demonstrated that Src is activated in TNBCs during the different stages of brain metastasis, particularly during BBB transmigration and brain parenchyma invasion. This work is in accordance with existing studies in the literature that found that Src inhibition significantly suppressed transmigration of breast cancer cells through the BBB [23]. In this work, we observed that Src activation may be linked to the formation of the TrkA/EphA2 complex induced by proNGF. Moreover, we observed that this activation depends on TrkA phosphorylation and on the recruitment of EphA2, which supports this activation in the absence of TrkA phosphorylation. These results are in line with the fact that EphA2 induces Src in cancers to support invasive migration [47].

The knowledge of key steps that occur in the brain metastasis process would have major implications for the design of improved therapies, which are still ineffective to date. Many factors, such as the mechanism of extravasation [48], angiogenesis [49] and tumoral persistence [50], are still a challenge. Indeed, our knowledge of tumoral persistence within TNBC remains obscure, in part due to the lack of a relevant in vitro study model. For these reasons, in this study, we used multiple models to evaluate the distinct aspects of the tumor microenvironment. These aspects include the cellular component, where brain endothelial cells and glial cells are known to be key players in intercellular communication between tumor cells and promote metastatic progression [51]. Beyond this cellular component, the three-dimensional structure of the extracellular matrix and its composition modulate the phenotype of tumor cells as well as their migratory and invasive properties [52–54]. Both the composition and elasticity of the organotypic extracellular matrix (BIOMIMESYS®) are similar to those found in different organs, such as the brain and liver, which are preferential metastatic sites of TNBC cells [55]. Through this work, we showed a differential response to TrkA/EphA2 axis inhibitors depending on the nature of the extracellular matrix in which the cancer cells were cultured. Nevertheless, we still do not know the underlying mechanisms responsible for these differences. Modulating the physical/chemical properties to determine whether the composition and/or the structure of the extracellular matrix have a specific effect could be an interesting approach to better understanding these responses [56]. Indeed, by modulating the elasticity of a hydrogel, Kondapaneni and Rao demonstrated that brain-derived MDA-MB-231 (MDA-MB-231 BR) cells cultured in matrices with low elasticity maintained a dormant phenotype [57]. Here, we showed that in MDA-MB-231 TrkA cells, inhibition of TrkA/EphA2 signaling also generated this phenotype. Thus, TrkA/EphA2 signaling would promote cell proliferation versus dormancy in very low elasticity matrices.

Breast cancer cells that metastasize to the brain have to develop new properties to cross the BBB. Recently, different molecular pathways that promote cancer cell transmigration across the BBB have been identified. For instance, metalloproteases such as MMP-9 [58] or MMP-2 [59] were found to be overexpressed in brain metastases of lung adenocarcinoma cells and breast cancer cells, respectively. These MMPs secreted by metastasizing tumor cells damage the integrity of the BBB by disrupting tight junctions shaped by Claudin-5, creating space for the invasion of cancer cells. Furthermore, some growth factors, such as VEGF, enhance the transendothelial migration process by promoting the adhesion of breast cancer cells to the endothelium and by disrupting VE-cadherin complexes that form tight junctions [60]. Our results indicated, for the first time, that TrkA and proNGF improve the transmigration of cancer cells across the BBB by increasing their ability to form membrane protrusions with activated Src. Although the mechanisms by which cancer cells transmigrate across the BBB have been described as being either paracellular [61, 62] or transcellular [62], the key players in TNBC extravasation are not yet fully understood. The process of BBB transmigration requires dynamic changes in cancer cell shape, as well as the formation of specific protrusive structures that facilitate invasion through the blood vessel [63]. The lack of understanding of the mechanisms and scarcity of accurate models to study metastasis of TNBC are major challenges for the development of effective TNBC treatments. To date, systemic treatments or targeted therapies have shown limited efficacy on metastatic cancers, with a limited increase in survival (5 months) for Troveldy, specifically [64]. Therefore, our study models and the results obtained from using them are of major importance in the development of effective anti-metastatic therapies.

## Conclusion

By recapitulating the final key steps of brain metastasis using 3D in vitro, ex vivo and in vivo models, we sought to better understand the underlying molecular mechanisms. In this work, we demonstrate the involvement of proNGF in the development of brain metastases. First, we identified the TrkA/EphA2 complex as a mediator of proNGF-induced BBB transmigration. Furthermore, our results showed that combined inhibition of TrkA and EphA2 significantly reduced brain metastasis in a preclinical breast cancer model. These results challenge the current understanding of the mechanisms of brain metastasis and highlight the role of proNGF as a key factor in the brain tropism of metastatic triple negative breast cancer.

### Supplementary Information

**Additional file 1: Figure S1**. proNGF increases TrkA/EphA2 complexes in TNBC regardless of p75NTR. Representative (**A**) pictures and associated quantification (**B**) of TrkA/EphA2 PLA signal following proNGF stimulation (60 min) in MDA-MB-231 TrkA treated with p75NTR targeting siRNA. Unpaired 2-tailed t test for B. ***P ≤ 0.001, ****P ≤ 0.0001, ns (non-significant). For A, scale bar = 10 µm. **Figure S2**. proNGF induces Src activation independently of TrkA phosphorylation in TNBC. Immunoblotting (**A**) and associated quantification (**B**) from MDA-MB-231 TrkA after proNGF stimulation (60 min) with Entrectinib (doses range from 1 to 500 nM) for TrkA, Src (and their phosphorylation form) and actin. (**C**) Immunoblotting for EphA2 from MDA-MB-231 TrkA treated with EphA2-targeting siRNA. **Figure S3**. proNGF induces Src activation regardless of p75NTR in TNBC. (**A-F**) Representative confocal images from MDA-MB-231 TrkA (**A**) and MDA-MB-231 HA-TrkA KD (**D**) of Src active form (Red) and EphA2 (Blue) staining with pSrc/EphA2 colocazation matrix with quantification associated for Src activity (B and E) and for EphA2/pSrc colocalization (C and F). Data in A-F are representative of 3 independents experiments on 30 cells/condition each. Data in B and E are presented with violin plots demonstrating the median (red bold line), quartiles (dots lines), variability and density. Data in C and E are represented by column bar graph with SD. Unpaired 2-tailed t test for B, C, E and F. *P ≤ 0.05, ***P ≤ 0.001, ****P ≤ 0.0001. For C, scale bare = 15 µm. **Figure S4**. Real time monitoring of Src activation induced by proNGF in TNBC cells. (**A**) Scheme of FRET Src reporter from Ouyang et al. that is characterized by an ECFP/YPET pair, an SH2 domain, a Src substrate domain and a prenylation site (22). (**B**) Emission ratio images and profiles along the indicated white lines of the ECFP/YPet-based Src biosensor in response to proNGF stimulation with or without prior transfection of siEphA2 on MDA-MB-231 TrkA KD. (**C-E**) Cellular-scale activity of Src is quantified by photobleaching of the FRET reporter acceptor (AB-FRET) in MDA-MB-231 TrkA KD (C & D) and MDA-MB-231 TrkA (E) after proNGF stimulation (60 min) with or without prior inhibitors treatments (siEphA2 and/or Entrectinib). Data in B-C are representative of 3 independents with 3 cells/conditions for A to C. Data in D and E are representative of 1 experiment with 3 cells/conditions. Data in B and D are represented by column bar graph with SD. One-way ANOVA followed by Dunnett’s test for B and D. **P ≤ 0.01, ***P ≤ 0.001, ns (non-significant). For A scale bar = 10 µm. **Figure S5**. ProNGF induced TNBC cells trans-BBB migration doesn't affect BBB integrity. (**A**) The permeability (Pe) coefficient of the endothelial layer is evaluated by the low-molecular-weight integrity marker Lucifer yellow (LY) on 3 µm pores insert after a 16 hours trans-BBB migration of MDA-MB-231 TrkA with or without proNGF abluminal stimulation and/or prior siEphA2 transfection. (**B**) Representative confocal images showing Claudin-5 or VE-Cadherin (white) and Hoechst (blue) signal on endothelium according to proNGF abluminal stimulation. Data in A to C are representative of 3 independents experiments with all condition realized in duplicate. Data in A are represented by column bar graph with SD. ns (non-significant). **Figure S6**. TrkA/EphA2 does not affect liver tissue invasion of TNBC. (**A-B**) Representative pictures (A) and associated quantification (B) from the monitoring of CellTracker™ stained MDA-MB-231 HA-TrkA KD mouse liver slices invasion. Data in B are presented as a column bar graph with SD. Unpaired 2-tailed t test for B. ns (non-significant). The dot line in A represent the limit of the Matrigel® plug. For A, scale bar = 100 µm. **Figure S7**. Combinatorial treatment with TrkA- and EphA2- targeting inhibitor does not affect tumoral development in TNBC cells maintained in live-like matrix. (**A**) Scanning electron microscopy picture of cut view from brain-like 3D ECM BIOMIMESYS®. (**B**) After 21 days of culture, the shape of fixed MDA-MB-21 WT/TrkA/TrkA KD are revealed with dapi (blue) and phalloidin (green) to allow cells and colonies segmentation after images acquisition with confocal microscope. (**C-H**) According to the TrkA overexpression status in MDA-MB-231, cell number (C & E) and colonies volumes (F - H) are quantified after entrectinib and/or EphA2 targeting siRNA of 21 days culture in 3D liver-like ECM. Unpaired 2-tailed t test for C, D, F and G. Two-way ANOVA followed by Tukey’s test for E and GH. **P ≤ 0.01, ns (non-significant). For B, scale bar = 300 µm. **Figure S8**. Combinatorial treatment with TrkA- and EphA2- targeting inhibitor delays tumor growth and does not affect mice health condition. After tumor relapse from MDA-MB-231 HA-TrkA were xenografted in SCID mice, mice are treated with Entrectinib and/or EphA2-targeting siRNA 5 times every 2-3 days. (**A**) Tumor volume was measured every 2-3 days. Mice weight were follow-up for each treatment (**B**). Data in A are represented by connecting line graph with mean ± SEM. Data in B are presented as points graph with median. Two-way ANOVA followed by Dunnet’s test for A was performed, the control condition was compared with entrectinib (a), siEPhA2 (b) or combinatorial treatment (c). One-way ANOVA followed by Tukey’s test for B. *P ≤ 0.05, ***P ≤ 0.001, ns (non-significant).

**Additional file 2: Video S1**: proNGF induce Src activation and relocalization in MDA-MB-231 regardless TrkA phosphorylation. Emission ratio images and profiles along the indicated white lines of the ECFP/YPet-based Src biosensor in response to proNGF stimulation with prior transfection of siCTRL on MDA-MB-231-TrkA-KD.

**Additional file 3: Video S2:** proNGF induce Src activation and relocalization in MDA-MB-231 trough TrkA/EphA2. Emission ratio images and profiles along the indicated white lines of the ECFP/YPet-based Src biosensor in response to proNGF stimulation with prior transfection of siEphA2 on MDA-MB-231-TrkA-KD.

**Additional file 4: Video S3: **Src activation is enhanced during BBB transmigration process in MDA-MB-231 (Luminal view). Emission ratio images of the ECFP/YPet-based Src biosensor during MDA-MB-231-TrkA-KD trans-BBB migration under abluminal proNGF stimulation (luminal side point of view).

**Additional file 5: Video S4: **Src activation is enhanced during BBB transmigration process in MDA-MB-231 (Abluminal view). Emission ratio images of the ECFP/YPet-based Src biosensor during MDA-MB-231-TrkA-KD trans-BBB migration under abluminal proNGF stimulation (abluminal side point of view).

**Additional file 6: Video 5: **Time lapse and cell tracking during brain slice invasion process. After dissection, the mouse brain is thickly sliced and cultured with the TNBC on an air/liquid interface. CellTracker™ stained MDA-MB-231 TrkA are segmented and classified according to their position (inside (red) or outside (white) the brain slice). Time is expressed in hours.

## Data Availability

The data and materials generated in this study are available upon reasonable request from the corresponding author.

## Abbreviations

BBB: Blood–Brain Barrier
EC: Endothelial Cell
KD: Kinase Dead
LY: Lucifer Yellow
(pro)NGF: (Pro)-Nerve Growth Factor
SCID: Severe Combined Immunodeficient
TrkA: Tropomyosin kinase A
TNBC: Triple Negative Breast Cancer
VEGF: Vascular Endothelial Growth Factor
