# Supplementary material for: ProNGF promotes brain metastasis through TrkA/EphA2 induced Src activation in triple negative breast cancer cells
Source: Exp Hematol Oncol. 2023 Dec 10;12:104. doi: 10.1186/s40164-023-00463-6 (PMC10710730; doi:10.1186/s40164-023-00463-6)
Supplement: Supplementary file 1 — Additional file 1: Figure S1. proNGF increases TrkA/EphA2 complexes in TNBC regardless of p75NTR. Representative (A) pictures and associated quantification (B) of TrkA/EphA2 PLA signal following proNGF stimulation (60 min) in MDA-MB-231 TrkA treated with p75NTR targeting siRNA. Unpaired 2-tailed t test for B. ***P ≤ 0.001, ****P ≤ 0.0001, ns (non-significant). For A, scale bar = 10 µm. Figure S2. proNGF induces Src activation independently of TrkA phosphorylation in TNBC. Immunoblotting (A) and associated quantification (B) from MDA-MB-231 TrkA after proNGF stimulation (60 min) with Entrectinib (doses range from 1 to 500 nM) for TrkA, Src (and their phosphorylation form) and actin. (C) Immunoblotting for EphA2 from MDA-MB-231 TrkA treated with EphA2-targeting siRNA. Figure S3. proNGF induces Src activation regardless of p75NTR in TNBC. (A-F) Representative confocal images from MDA-MB-231 TrkA (A) and MDA-MB-231 HA-TrkA KD (D) of Src active form (Red) and EphA2 (Blue) staining with pSrc/EphA2 colocazation matrix with quantification associated for Src activity (B and E) and for EphA2/pSrc colocalization (C and F). Data in A-F are representative of 3 independents experiments on 30 cells/condition each. Data in B and E are presented with violin plots demonstrating the median (red bold line), quartiles (dots lines), variability and density. Data in C and E are represented by column bar graph with SD. Unpaired 2-tailed t test for B, C, E and F. *P ≤ 0.05, ***P ≤ 0.001, ****P ≤ 0.0001. For C, scale bare = 15 µm. Figure S4. Real time monitoring of Src activation induced by proNGF in TNBC cells. (A) Scheme of FRET Src reporter from Ouyang et al. that is characterized by an ECFP/YPET pair, an SH2 domain, a Src substrate domain and a prenylation site (22). (B) Emission ratio images and profiles along the indicated white lines of the ECFP/YPet-based Src biosensor in response to proNGF stimulation with or without prior transfection of siEphA2 on MDA-MB-231 TrkA KD. (C-E) Cellula [file 40164_2023_463_MOESM1_ESM.pdf]

ProNGF promotes brain metastasis through TrkA/EphA2 induced Src activation in triple negative breast cancer cells

Julien Cicero, Sarah Trouvilliez, Martine Palma, Gaetan Ternier, Laurine Decoster, Eloise Happernegg, Nicolas Barois, Alexandre Van Outryve, Lucie Dehouck, Roland P Bourette, Eric Adriaenssens, Chann Lagadec, Cagatay Mehmet Tarhan, Dominique Collard, Zied Souguir, Elodie Vandenhaute, Grégory Maubon, François Sipieter, Nicolas Borghi, Fumitaka Shimizu, Takashi Kanda, Paolo Giacobini, Fabien Gosselet, Nathalie Maubon, Xuefen Le Bourhis, Isabelle Van Seuningen, Caroline Mysiorek and Robert-Alain Toillon.

Supplemental data

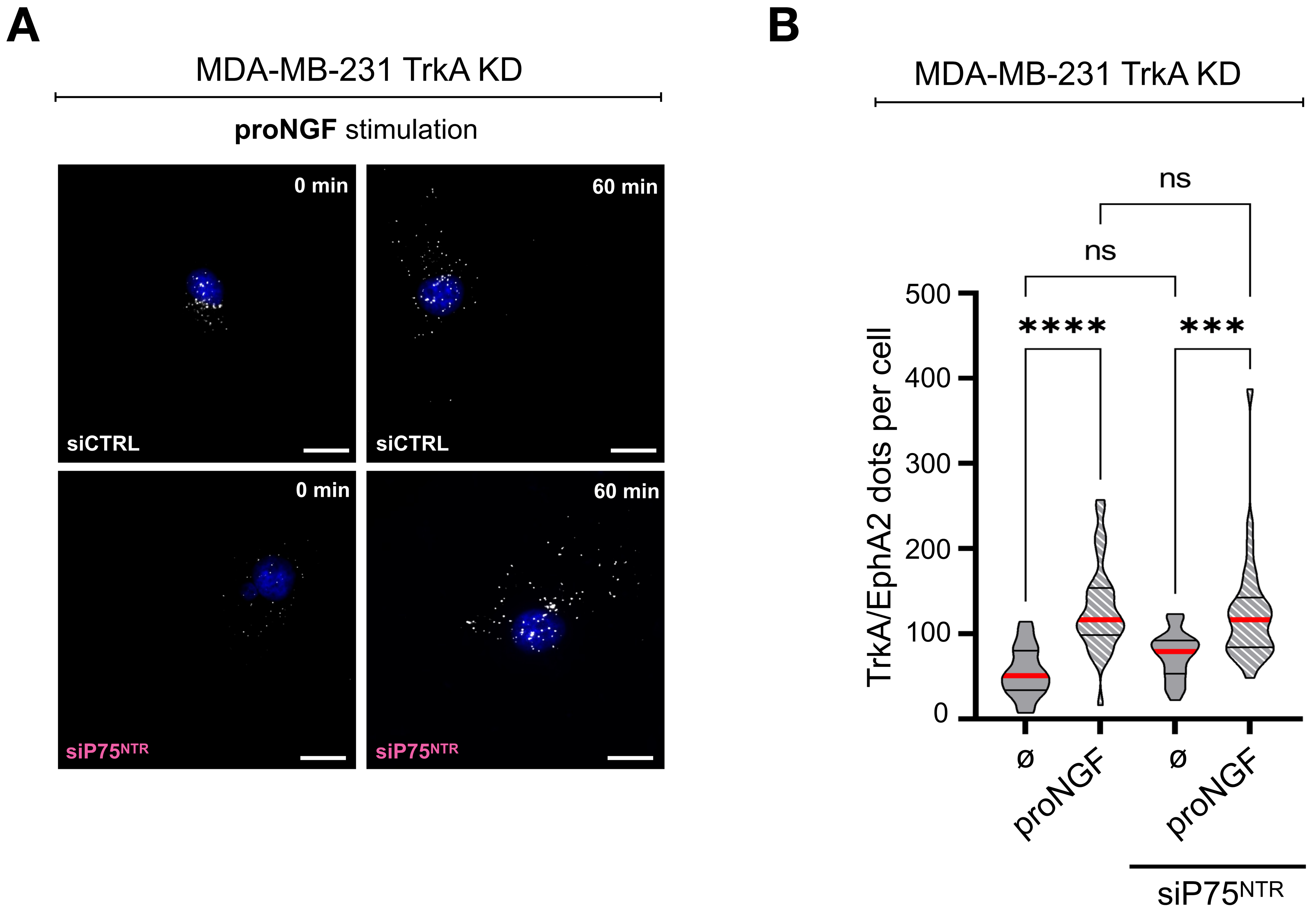
A
MDA-MB-231 TrkA KD
proNGF stimulation
0 min
60 min
siCTRL
siCTRL
0 min
60 min
siP75NTR
siP75NTR
B
MDA-MB-231 TrkA KD
ns
ns
****
***
TrkA/EphA2 dots per cell
500
400
300
200
100
0
ø
proNGF
ø
proNGF
siP75NTR

Figure S1.

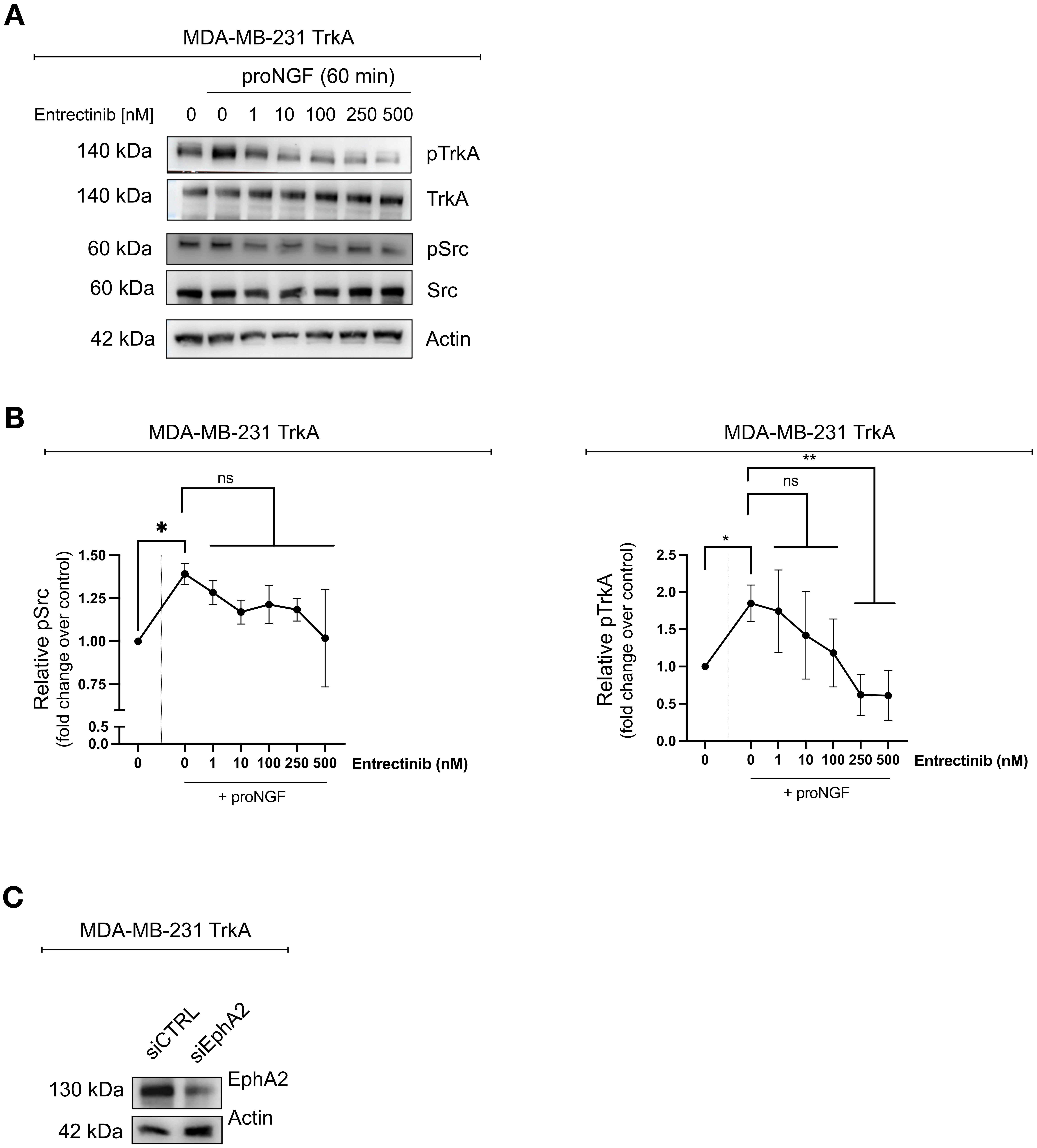
A
MDA-MB-231 TrkA
proNGF (60 min)
Entrectinib [nM] 0 0 1 10 100 250 500
140 kDa pTrkA
140 kDa TrkA
60 kDa pSrc
60 kDa Src
42 kDa Actin
B
MDA-MB-231 TrkA
Relative pSrc (fold change over control)
ns
*
1.50
1.25
1.00
0.75
0.5
0.0
0 0 1 10 100 250 500 Entrectinib (nM)
+ proNGF
MDA-MB-231 TrkA
Relative pTrkA (fold change over control)
**
ns
*
2.5
2.0
1.5
1.0
0.5
0.0
0 0 1 10 100 250 500 Entrectinib (nM)
+ proNGF
C
MDA-MB-231 TrkA
siCTRL
siEphA2
130 kDa EphA2
42 kDa Actin

Figure S2.

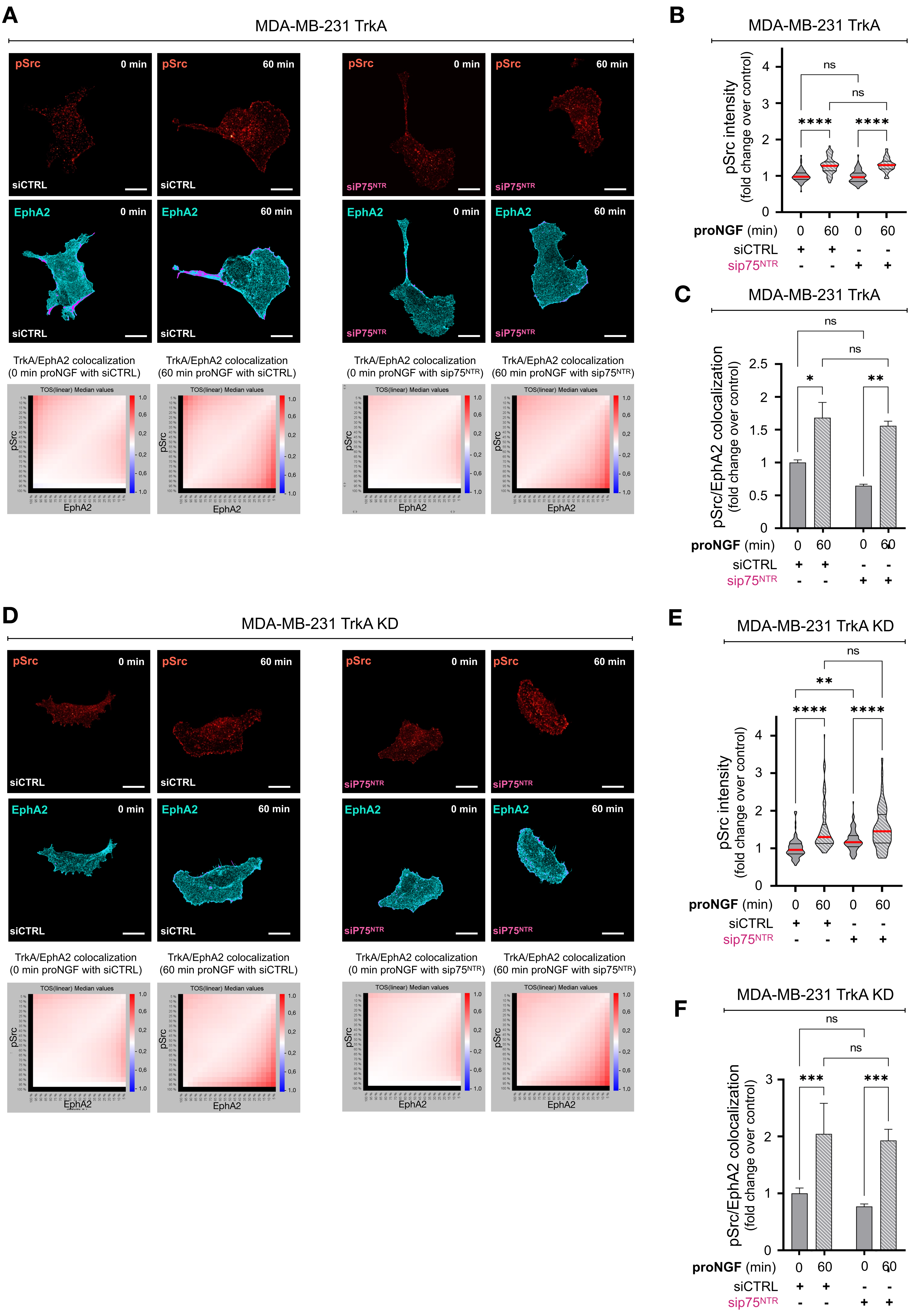

A
MDA-MB-231 TrkA
pSrc
0 min
60 min
EphA2
siCTRL
siP75NTR
TrkA/EphA2 colocalization
(0 min proNGF with siCTRL)
(60 min proNGF with siCTRL)
(0 min proNGF with sip75NTR)
(60 min proNGF with sip75NTR)
TOS(linear) Median values
B
MDA-MB-231 TrkA
pSrc intensity
(fold change over control)
ns
****
proNGF (min)
siCTRL
sip75NTR
C
MDA-MB-231 TrkA
pSrc/EphA2 colocalization
(fold change over control)
ns
*
**
D
MDA-MB-231 TrkA KD
E
MDA-MB-231 TrkA KD
F
MDA-MB-231 TrkA KD
***

Figure S3.

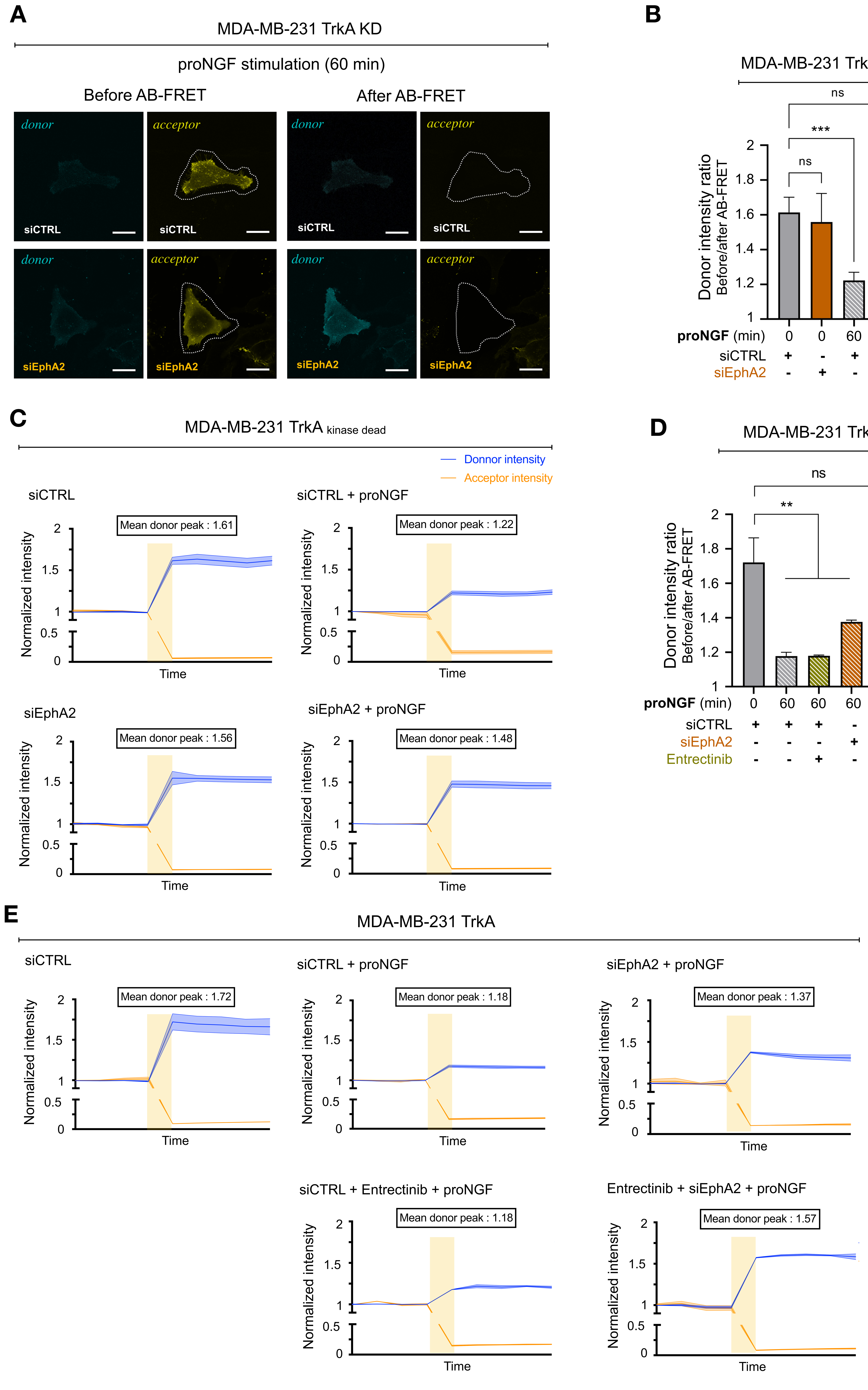
A
MDA-MB-231 TrkA KD
proNGF stimulation (60 min)
Before AB-FRET
After AB-FRET
donor
acceptor
siCTRL
siEphA2
B
MDA-MB-231 TrkA KD
Donor intensity ratio
Before/after AB-FRET
ns
***
proNGF (min)
siCTRL
siEphA2
C
MDA-MB-231 TrkA kinase dead
Donnor intensity
Acceptor intensity
siCTRL
siCTRL + proNGF
siEphA2
siEphA2 + proNGF
Mean donor peak : 1.61
Mean donor peak : 1.22
Mean donor peak : 1.56
Mean donor peak : 1.48
Normalized intensity
Time
D
MDA-MB-231 TrkA
Donor intensity ratio
Before/after AB-FRET
ns
**
proNGF (min)
siCTRL
siEphA2
Entrectinib
E
MDA-MB-231 TrkA
siCTRL
siCTRL + proNGF
siEphA2 + proNGF
siCTRL + Entrectinib + proNGF
Entrectinib + siEphA2 + proNGF
Mean donor peak : 1.72
Mean donor peak : 1.18
Mean donor peak : 1.37
Mean donor peak : 1.18
Mean donor peak : 1.57
Normalized intensity
Time

Figure S4

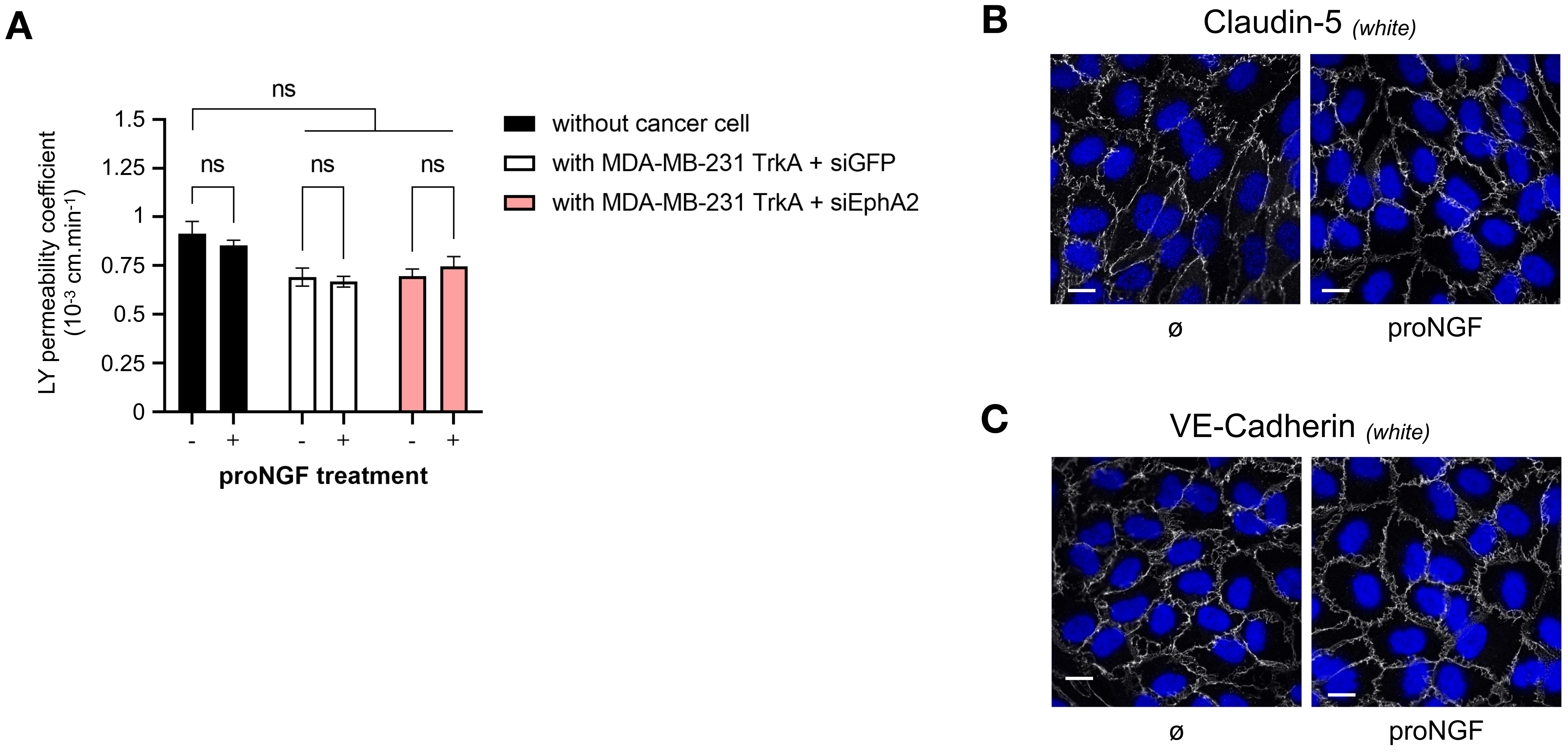
A
ns
ns
ns
ns
1.5
1.25
1
0.75
0.5
0.25
0
LY permeability coefficient
(10^{-3} cm.min^{-1})
- + - + - +
proNGF treatment
without cancer cell
with MDA-MB-231 TrkA + siGFP
with MDA-MB-231 TrkA + siEphA2
B
Claudin-5 (white)
ø
proNGF
C
VE-Cadherin (white)
ø
proNGF

Figure S5.

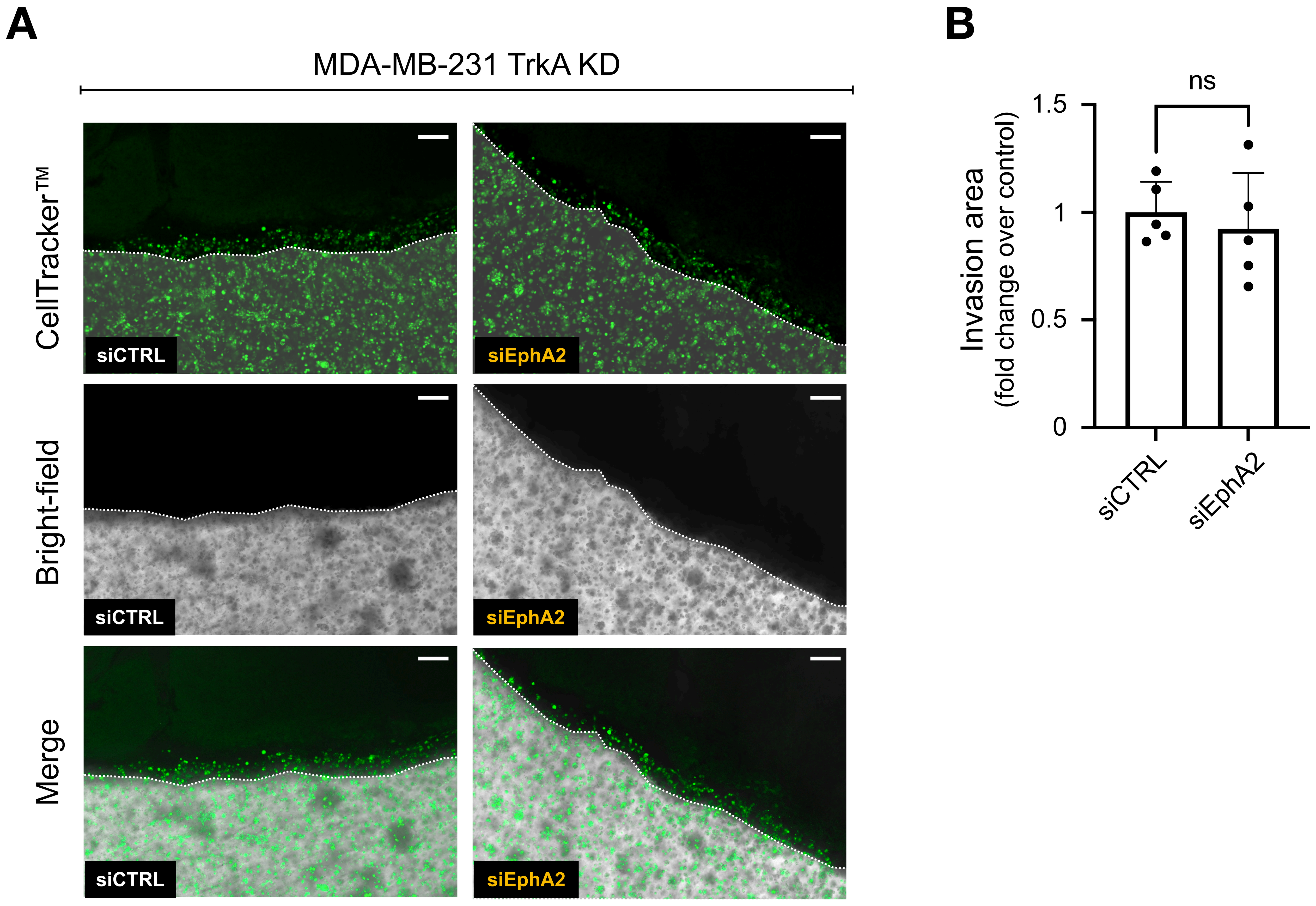
A
MDA-MB-231 TrkA KD
CellTracker™
Bright-field
Merge
siCTRL
siEphA2
B
ns
Invasion area
(fold change over control)
1.5
1
0.5
0
siCTRL
siEphA2

Figure S6.

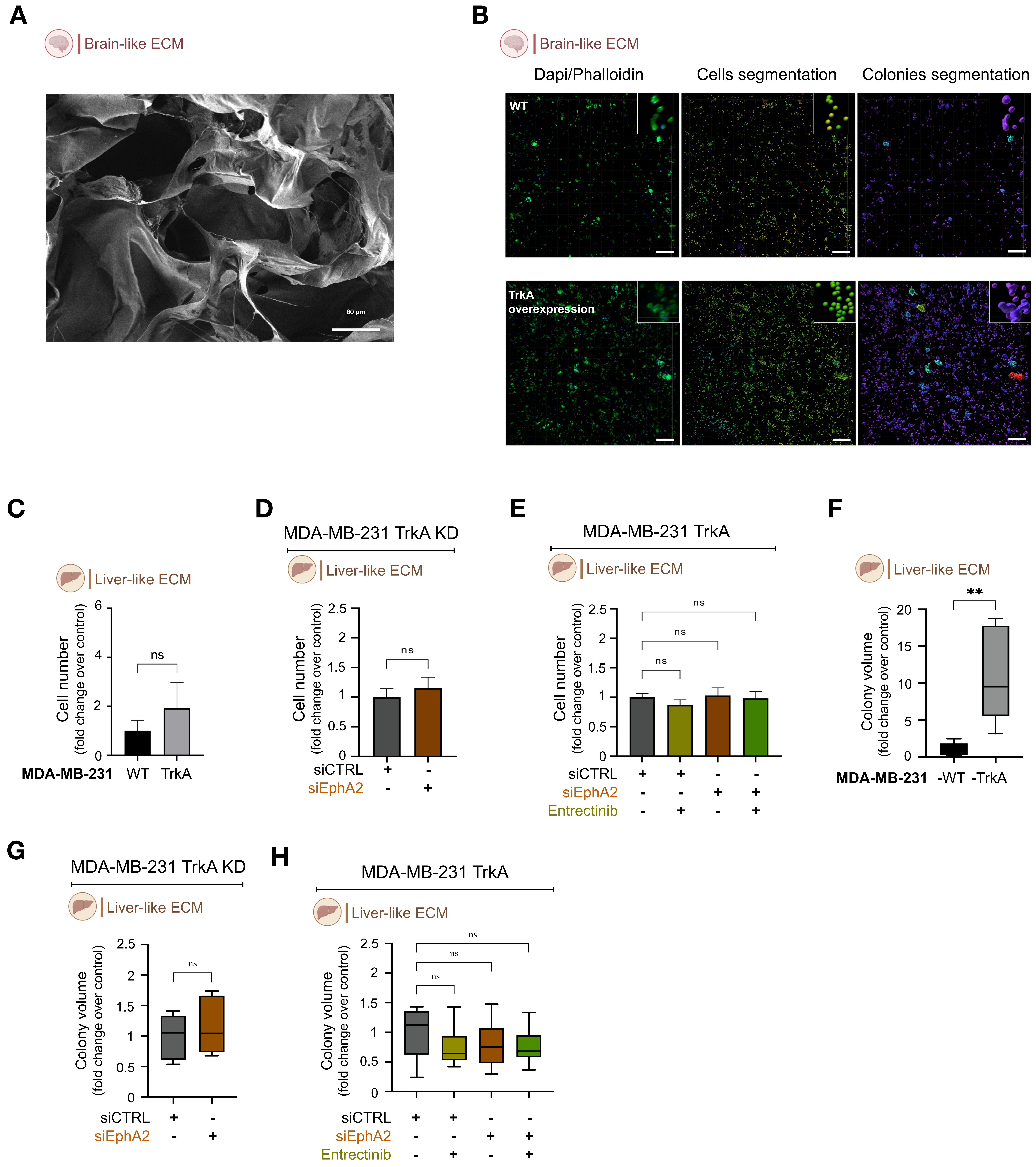

A
Brain-like ECM
80 µm
B
Brain-like ECM
Dapi/Phalloidin
Cells segmentation
Colonies segmentation
WT
TrkA overexpression
C
Liver-like ECM
Cell number (fold change over control)
ns
MDA-MB-231 WT TrkA
D
MDA-MB-231 TrkA KD
Liver-like ECM
Cell number (fold change over control)
ns
siCTRL + -
siEphA2 - +
E
MDA-MB-231 TrkA
Liver-like ECM
Cell number (fold change over control)
ns
siCTRL + + - -
siEphA2 - - + +
Entrectinib - + - +
F
Liver-like ECM
Colony volume (fold change over control)
**
MDA-MB-231 -WT -TrkA
G
MDA-MB-231 TrkA KD
Liver-like ECM
Colony volume (fold change over control)
ns
siCTRL + -
siEphA2 - +
H
MDA-MB-231 TrkA
Liver-like ECM
Colony volume (fold change over control)
ns
siCTRL + + - -
siEphA2 - - + +
Entrectinib - + - +

Figure S7

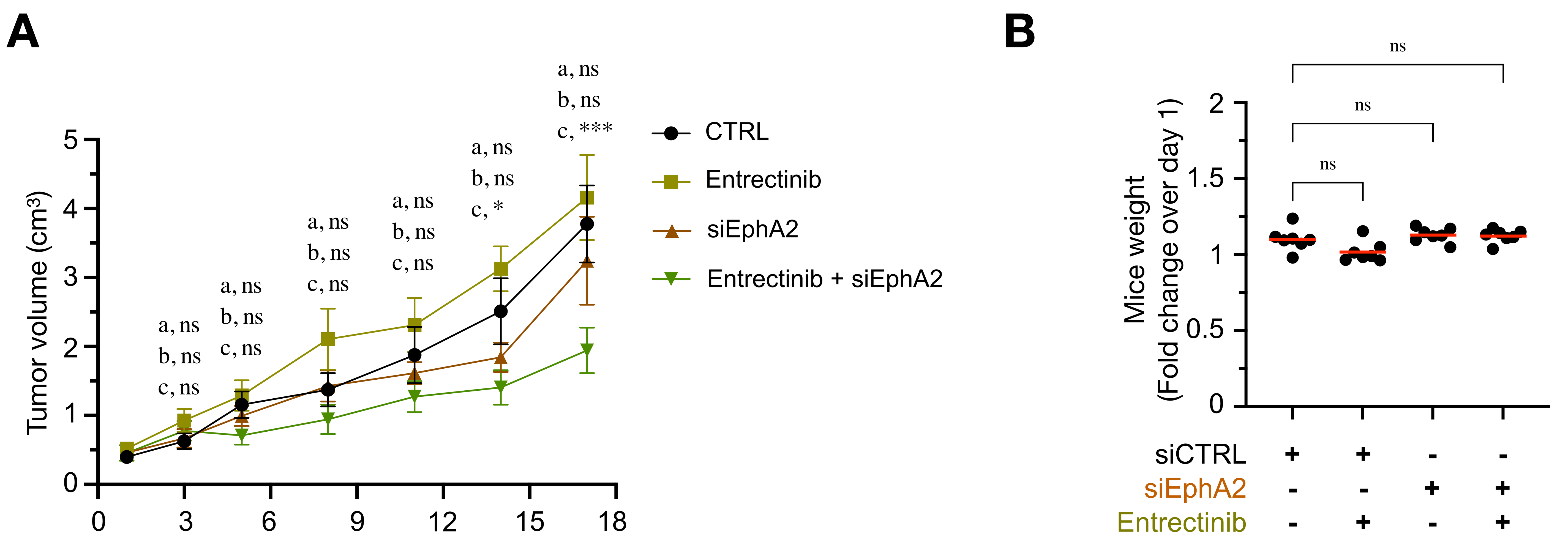
A
B
Tumor volume (cm³)
0
1
2
3
4
5
0
3
6
9
12
15
18
a, ns
b, ns
c, ns
a, ns
b, ns
c, *
a, ns
b, ns
c, ***
CTRL
Entrectinib
siEphA2
Entrectinib + siEphA2
Mice weight
(Fold change over day 1)
ns
siCTRL + + - -
siEphA2 - - + +
Entrectinib - + - +

Figure S8
